# The HAPPE plus Event-Related (HAPPE+ER) software: A standardized preprocessing pipeline for event-related potential analyses

**DOI:** 10.1016/j.dcn.2022.101140

**Published:** 2022-07-19

**Authors:** A.D. Monachino, K.L. Lopez, L.J. Pierce, L.J. Gabard-Durnam

**Affiliations:** a Northeastern University, 360 Huntington Ave, Boston, MA, United States; b York University, 4700 Keele Street, Toronto, ON, Canada

**Keywords:** Electroencephalography, Processing pipeline, Event-related potential, ERP, HAPPE, HAPPE+ER, Automated preprocessing, Wavelet, Simulated ERP

## Abstract

Event-Related Potential (ERP) designs are a common method for interrogating neurocognitive function with electroencephalography (EEG). However, the traditional method of preprocessing ERP data is manual-editing – a subjective, time-consuming processes. A number of automated pipelines have recently been created to address the need for standardization, automation, and quantification of EEG data pre-processing; however, few are optimized for ERP analyses (especially in developmental or clinical populations). We propose and validate the HAPPE plus Event-Related (HAPPE+ER) software, a standardized and automated pre-processing pipeline optimized for ERP analyses across the lifespan. HAPPE+ER processes event-related potential data from raw files through preprocessing and generation of event-related potentials for statistical analyses. HAPPE+ER also includes post-processing reports of both data quality and pipeline quality metrics to facilitate the evaluation and reporting of data processing in a standardized manner. Finally, HAPPE+ER includes post-processing scripts to facilitate validating HAPPE+ER performance and/or comparing to performance of other preprocessing pipelines in users’ own data via simulated ERPs. We describe multiple approaches with simulated and real ERP data to optimize pipeline performance and compare to other methods and pipelines. HAPPE+ER software is freely available under the terms of GNU General Public License at https://www.gnu.org/licenses/#GPL

## 1 Introduction

There is growing momentum to standardize and automate electroencephalography (EEG) and event-related potential (ERP) pre-processing to meet the needs of contemporary electrophysiological studies. Until recently, the traditional method of preparing EEG/ERP data for analysis involved removing artifact-laden segments through subjective manual editing. However, this process can result in significant data loss, especially in data from developmental and clinical populations characterized by high levels of artifact. This process has also become difficult to scale as sample sizes and electrode densities for recording have both increased substantially over the last decade. Furthermore, the subjective nature of manual editing impedes comparisons across EEG acquisition systems, datasets, and laboratories. The solution appears in automated, standardized processing. However, software was often limited to single stages of EEG pre-processing, like line-noise removal (e.g., CleanLine (Mullen, 2012)) or automated ICA component rejection (i.e., MARA (Winkler et al., 2011), ADJUST (Mognon et al., 2011)); or was developed only on data with low levels of artifact; and lacked imbedded metrics to quantitatively assess their performance or data quality. HAPPE software (Gabard-Durnam et al., 2018) proposed a solution to these limitations by providing an automated, quantifiable, and standardized method of processing EEG data that is effective with high levels of artifact as seen in developmental and clinical populations.

HAPPE is not alone in its endeavors; a growing number of pipelines, scripts, and software now also address the need for standardized pre-processing methods for EEG data. With the breadth of available tools comes a diversity of approaches to EEG data processing (Bigdely-Shamlo et al., 2015, Cassani et al., 2017, da Cruz et al., 2018, Debnath et al., 2020, Desjardins et al., 2021, Gabard-Durnam et al., 2018, Gramfort et al., 2014, Hatz et al., 2015, Leach et al., 2020, Oostenveld et al., 2011, Pedroni et al., 2019, Tadel et al., 2011). Few empirical comparisons have been made between pipelines, and it may be difficult for researchers to assess which pipeline works best on their EEG data. Moreover, pipelines differ in their limitations on the kinds of analyses that can be performed post-processing. Some pipelines are restricted to preparing data for time-frequency analyses or resting-state EEG data (Cassani et al., 2017, Gabard-Durnam et al., 2018, Hatz et al., 2015). Pipelines also differ in the populations for which they have been validated. Namely, the majority of pipelines are tested and validated using data from healthy adults (Bigdely-Shamlo et al., 2015, Cassani et al., 2017, da Cruz et al., 2018, Mognon et al., 2011, Nolan et al., 2010, Pedroni et al., 2019). Far fewer specify compatibility and validation with developmental or clinical populations, whose data often have physiological and acquisition differences from healthy adult data. Exceptions include MADE (Debnath et al., 2020) (validated in developmental data); EEG-IP-L (Desjardins et al., 2021), and HAPPE (Gabard-Durnam et al., 2018) (validated in clinical and developmental data). The optimal pipeline would offer validated solutions suitable to developmental, clinical, and adult EEG/ERP pre-processing needs to facilitate comparisons across studies and ages.

Here we propose and validate the HAPPE plus ER (HAPPE+ER) software to address these limitations for ERP pre-processing, improve on the original HAPPE pre-processing strategies, and increase accessibility across acquisition setups and user coding fluencies. ERP analysis is a common approach to characterize EEG data by examining the time-locked neural responses to stimuli recorded during task paradigms (Herrmann and Knight, 2001). As such, ERPs are precise temporal representations of neural activity. Moreover, different components of the time-locked ERP waveforms reflect disparate sensory, perception, affective, and cognitive phenomena in the brain (Herrmann and Knight, 2001, Klawohn et al., 2020, Lopez-Calderon and Luck, 2014). To facilitate automated, standardized processing of EEG data for ERP analyses across the lifespan, HAPPE+ER includes both code to pre-process ERP data and code that enables the efficient, automated creation of processed ERP figures and measures, including (but not limited to) peak and mean amplitudes, latencies, and area under the curve (though we note our focus is primarily pre-processing given the excellent tools available for sophisticated ERP visualization and quantification like ERPLAB (Lopez-Calderon and Luck, 2014), EEGLAB (Delorme and Makeig, 2004), and ERA Toolbox (Clayson and Miller, 2017)). HAPPE+ER (pronounced “happier”) incorporates pre-processing steps for ERP designs that we validate in adult, developmental, and clinical data. HAPPE+ER also introduces a novel pipeline quality report of metrics reflecting each pre-processing step’s performance on ERP data to aid researchers in assessing whether the pipeline is effectively pre-processing their data. That is, HAPPE+ER now includes complementary, quantifiable measures of quality for both data inputs (data quality report) and processing methods (pipeline quality report). In conjunction with these changes, data can be input from an increased array of file formats and acquisition layouts, including caps from EGI, BioSemi, and Brain Products. These changes greatly expand the breadth of data for which HAPPE+ER is suitable while maintaining standardization across a variety of input parameters and output analyses, rendering HAPPE+ER a flexible software suited to electrophysiological processing across the lifespan and populations.

**Fig. 1:**
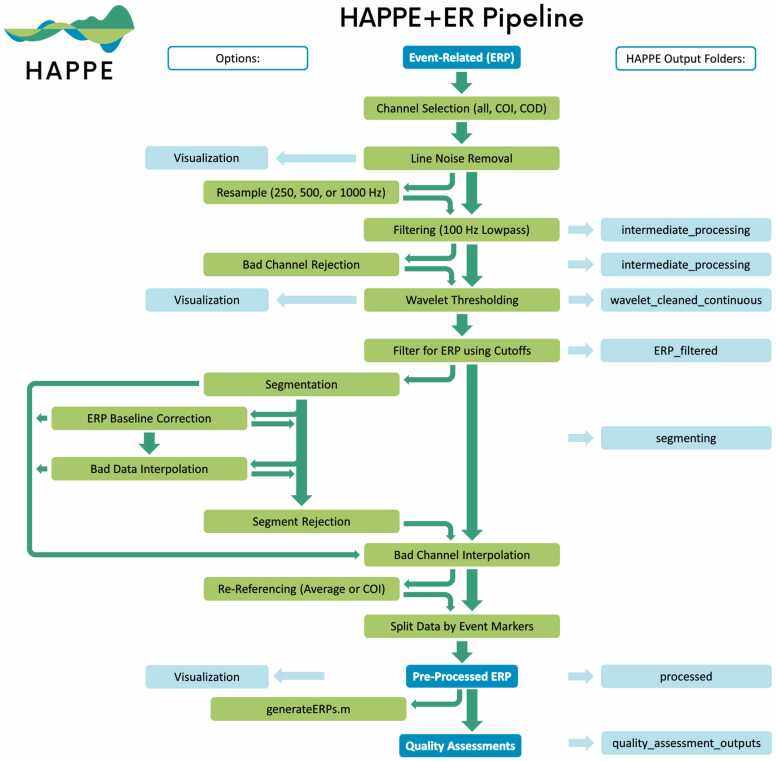
Image illustrating the HAPPE+ER pipeline’s processing steps. Intermediate outputs are noted by the light blue boxes on the right and are labeled according to the folder where they are saved. User options are displaced to the left, with bright green arrows indicating all possible methods of flow between options.

The following sections first detail and justify the HAPPE+ER pipeline’s pre-processing steps for ERP analyses and outline HAPPE+ER’s quality control metrics (Fig. 1). Second, we compare HAPPE+ER’s approach to artifact correction with multiple alternative approaches in simulated ERP data (comparisons in real adult, developmental, and clinical ERP data available in supplements). Third, we evaluate HAPPE+ER’s effectiveness as a pipeline with ERP datasets relative to other automated pipelines. Finally, we outline how to access HAPPE+ER software and the datasets included in this manuscript as freely-available public resources.

## 2 HAPPE+ER pipeline steps

Fig. 1 HAPPE+ER pipeline schematic.

### 2.1 HAPPE+ER data inputs

HAPPE+ER accommodates multiple types of EEG files with different acquisition layouts as inputs, with additional options on top of those previously supported in HAPPE 1.0 software. See Table 1 for complete layout and formatting options accepted by the HAPPE+ER pipeline. For.set formatted files, the correct channel locations should be pre-set and embedded in the file (e.g., by loading it into EEGLAB (Delorme and Makeig, 2004) and confirming the correct locations) prior to running through HAPPE+ER. Each batch run of HAPPE+ER must include files collected with the same channel layout (company, acquisition layout/cap, and electrode number) and paradigm (resting-state or event-related), each of which users must specify for a given run. The same is true of file formats, in that a single run will support only a single file type across files, specified by the user. HAPPE+ER processes data collected with any sampling rate, and files within a single run may differ in their individual sampling rates.

**Table 1:** A list of acquisition layouts and associated file types supported by HAPPE+ER. Includes company name and number of channels/leads.

Company	Net Name	Number of Channels	Supported File Types
Magstim EGI	Geodesic Sesnor Net	64	.mat (NetStation & MATLAB matrix),.raw,.set,.mff
	HydroCel Geodesic Sensor Net	32, 64, 128, 256	.mat (NetStation & MATLAB matrix),.raw,.set,.mff
Neuroscan	Quik-Cap	32, 64, 128, 256	.cdt,.set,.mat (MATLAB matrix)
Mentalab		1–16	.edf
Other Low-Density Layouts		~32 or fewer	.set,.mat (MATLAB matrix)
Other High-Density Layouts		~33 or more	.set,.mat (MATLAB matrix)

Stimuli markers, paradigm events, and/or conditions of interest (generally referred to as event markers moving forward) should be present in the data read into HAPPE+ER. How event markers become embedded in the data file will be stimulus-presentation software and acquisition system dependent. For some systems (e.g., EGI), native software facilitates translating event tracks (dropped by stimulus-presentation software) into conditions and trials marked within the data that HAPPE+ER (and other software) can read. HAPPE+ER relies on EEGLAB functions for reading in user data, so if EEGLAB registers a user’s event markers (which can quickly be verified through their GUI, please see HAPPE+ER user guide for further detail), HAPPE+ER will also be able to read those same event markers. If a user is unsure of their event marker names in the data, they can also use the EEGLAB GUI to verify event marker names before running HAPPE+ER on the data. Users may enter the full list of event marker types found across the dataset files, even if a single file does not contain all of those event types (e.g. participant did not complete the paradigm, did not generate a specific condition of interest via responses, etc.). Users do not need to know a priori which trial types exist in which files, either. HAPPE+ER outputs include all user-input stimuli conditions and will note that no trials for that condition(s) were detected in that specific file if this is the case. Similarly, users may also input a subset of the available event marker types within their files if desired, though only those user-input event marker types will be available for further HAPPE+ER processing (e.g. segmentation, baseline correction) and in HAPPE+ER outputs. Please note that while HAPPE+ER does not currently support.mat formats with separate event lists, this functionality is in progress for the next HAPPE+ER update.

### 2.2 Channel selection (all, channels of interest (COI), channels of disinterest (COD))

HAPPE+ER offers a variety of options for channel selection such that the user can choose the channels that best fit the needs of their dataset. HAPPE+ER does not restrict the user to a specific number of channels, as no later processing steps rely on channel number for robust processing. Specifically, HAPPE+ER supports the following options: (1) Selecting all channels, which results in each channel included in the dataset being included in the following processing steps. (2) Alternatively, the user can select a subset of channels of interest, which can be selected via inclusion (channels of interest, COI) or exclusion (channels of disinterest, COD) methods.

Selecting the option to include user-specified channels will remove every channel not included in the user-specified list from subsequent processing with the inability to recover them later. For example, for data from a 128-channel cap where the user selects 20 channels, the post-HAPPE+ER processed data will contain only data for those 20 selected channels.

Selecting the option to exclude channels does the opposite, excluding all user-specified channels and keeping those not included in the user-specified list. For example, for data from a 128-channel cap where the user specifies 8 channels, the post HAPPE+ER processed data will contain the remaining 120 channels. These options increase the ease of selecting channels based on the number of channels a user is interested in examining.

### 2.3 Line noise removal

HAPPE+ER removes electrical noise (e.g., 60 or 50 Hz artifact signal) from EEG through the multi-taper regression approach implemented by the CleanLine program (Mullen, 2012). Multi-taper regression can remove electrical noise without sacrificing or distorting the underlying EEG signal in the nearby frequencies, drawbacks of the notch-filtering approach to line-noise removal (Mitra and Pesaran, 1999). Specifically, HAPPE+ER applies the updated version of CleanLine’s multi-taper regression which is more effective at removing line noise than the original version present in HAPPE 1.0. The legacy CleanLine version from HAPPE 1.0 is available as an option to the user, however the updated version is registered as the default. CleanLine’s multi-taper regression scans for line-noise signal near the user-specified frequency ± 2 Hz, a 4-s window with a 1-s step size and a smoothing tau of 100 during the fast Fourier transform, and a significance threshold of p = 0.01 for sinusoid regression coefficients during electrical noise removal. This process is highly specific to the frequency of electrical noise, which the user can specify to be 60 Hz or 50 Hz. The user may also specify harmonics to reduce if there is excessive line noise in additional frequencies (e.g. 30 Hz, 25 Hz). Pipeline quality control metrics for the effectiveness of line noise removal are automatically generated in HAPPE+ER and discussed in detail as part of the subsequent “Quality Control Metrics” section of this manuscript.

### 2.4 Resample (250, 500, or 1000 Hz) (optional)

Users may optionally choose to resample their data to 250, 500, or 1000 Hz. Users may use this option to reduce file sizes or to align data with other projects or files collected at a lower sampling rate (note, users may not upsample data, e.g. from 500 to 1000 Hz). As HAPPE+ER functionality is optimized for these common sampling rates (e.g. wavelet-thresholding steps described below are optimized for data around these sampling rates), users with sampling rates far from any of these options may achieve optimal performance of HAPPE+ER by resampling (e.g. from 2000 Hz down to 1000 Hz).

### 2.5 Filtering (100 Hz lowpass)

HAPPE+ER applies an automatic low-pass filter at 100 Hz prior to artifact rejection and bad channel detection (if selected) so these steps can evaluate data within the frequency range of neural activity. Additional filtering for the specific ERP(s) of interest as determined by the user occurs after artifact rejection. Note this filtering strategy differs from that in HAPPE 1.0, where a 1 Hz high-pass filter was applied to all files to facilitate optimal ICA decomposition (Winkler et al., 2015). HAPPE 1.0 precluded ERP analyses since filtering at 1 Hz excludes frequencies of interest in ERP analyses.

### 2.6 Bad channel rejection (optional)

HAPPE+ER can detect and remove channels that do not contribute to usable brain data due to high impedances, damage to the electrodes, insufficient scalp contact, and excessive movement or electromyographic (EMG) artifact throughout the recording. HAPPE+ER performs the following steps (with thresholds determined by empirical optimization and justified below) over the entire data file-length submitted for processing:

1. Detect flat-line channels (via Clean RawData function; reject if flat > 5 s)
2. Detect outlier channels based on their power spectrum (via EEGLAB rej_chan function run twice; reject if > 3.5 standard deviations or < −5 standard deviations from mean power)
3. Detect remaining overwhelming line noise contamination (via Clean RawData’s line noise criterion; reject if > 6 standard deviations from mean line noise/neural signal ratio)
4. Detect outlier channels based on correlation with all other channels (via Clean RawData’s channel criterion; reject if < 0.8 correlation with other channels)

HAPPE+ER performs bad channel detection that is suitable for ERP data and expands the classes of bad channels that can be detected relative to HAPPE 1.0 by combining EEGLAB’s Clean Rawdata functions with power spectral evaluation steps as follows. First, HAPPE+ER runs the Clean Rawdata ‘Flatline Criterion’ to detect channels with flat recording lengths longer than 5 s, indicating no data collected at that location. If the channel contains a flatline that lasts longer than 5 s, the channel is marked as bad.

After flat channels have been removed, HAPPE+ER includes a spectrum-based bad channel detection step similar to that used in HAPPE 1.0 where bad channel detection is achieved by twice evaluating the normed joint probability of the average log power from 1 to 125 Hz across the user-specified subset of included channels. Channels whose probability falls more than 3 standard deviations from the mean are removed as bad channels. While the HAPPE 1.0 method of legacy detection proved to be suboptimal for our test dataset (see Table 2), evaluating the joint probability of average power from 1 to 100 Hz was useful for optimizing bad channel detection alongside Clean Rawdata. HAPPE+ER thus includes a spectrum evaluation step with thresholds of − 5 and 3.5 standard deviations. Here, the standard deviations are not symmetric as artifact in the EEG signal mostly produces power spectrums with positive standard deviations from the mean. Channels near the reference electrode (e.g. roughly within 1″ of the reference electrode, in EGI nets, mostly channels in the nearest ring around online reference Cz) will have reduced amplitudes by virtue of sharing variance with the reference, rather than due to artifact, but will score below the mean in their average log power accordingly. To avoid falsely rejecting (good) channels near the reference electrode(s), the negative standard deviation threshold is set liberally (but arrived at through empirical testing) at –5 standard deviations. We note that the lenient lower standard deviation criteria (−5) for identifying outlier power spectra, while empirically part of the best-performing parameter combination, may allow low-amplitude (but greater than flat-line) bad channels through as good channels. This risk was evaluated (in our test dataset we did not observe any of these instances) and determined to be preferable to the observed regular exclusion of (good) low-amplitude channels near the user’s reference electrode with more conservative standard deviation criteria. Users should bear this risk in mind when evaluating their data.

**Table 2:** Performance of bad channel parameters tested on twenty files from an EGI dataset. The settings for each individual step are separated by a newline within the cell.

Bad Channel Parameters	Accuracy (780 Total Channels)	False Positive (741 Good Channels)	False Negatives (39 Bad Channels)
HAPPE 1.0 Legacy Detection	92.4%	41	18
Flatline = 5; Channel Corr = 0.1; Line Noise Ratio = 20 Spectrum = −5, 3.5 Channel Corr = 0.8; Line Noise Ratio = 6	97.6%	9	10
Flatline = 5; Channel Corr = 0.1; Line Noise Ratio = 20 Spectrum = −5, 3.5 Channel Corr = 0.75; Line Noise Ratio = 6	97.2%	7	15
Flatline = 5; Channel Corr = 0.1; Line Noise Ration = 20 Spectrum = −5, 3.5 Channel Corr = 0.8; Line Noise Ratio = 5	97.3%	11	10
Flatline = 5; Channel Corr = 0.1; Line Noise Ratio = 20 Spectrum = −4, 3.5 Channel Corr = 0.8, Line Noise Ratio = 6	97.2%	12	10
Flatline = 5; Channel Corr = 0.1; Line Noise Ratio = 20 Spectrum = −6, 3.5 Channel Corr = 0.8; Line Noise Ratio = 6	97.6%	9	10

Finally, HAPPE+ER uses Clean Rawdata’s ‘Line Noise Criterion’ and ‘Channel Criterion’ to detect additional bad channel cases. The line noise criterion identifies whether a channel has more line noise relative to neural signal than a predetermined value in standard deviations based on the total channel population, set in HAPPE+ER to 6 standard deviations. Channel criterion sets the minimally acceptable correlation value between the channel in question and all other channels. If a channel’s average correlation is less than the preset value, it is considered abnormal and marked as bad. HAPPE+ER uses a correlation threshold of.8 to identify bad channels through this approach.

Note that bad channel detection is performed over the entire recording being processed via HAPPE+ER. In the event that users include breaks between tasks with participant activity (e.g. stretching, talking) or have periods of non-interest in the recording session and have concern about channel artifacts during those windows that would cause channels to be flagged as bad during pre-processing, there are several approaches to achieve optimal bad channel detection performance in HAPPE+ER. For example, they may segment out the recording periods of interest (e.g. specific paradigms) as the file inputs to HAPPE+ER. Alternatively, users may start and stop the EEG recording during data collection to prevent artifact-laden data collection during non-paradigm periods. Thus, options to optimize file inputs to HAPPE+ER exist for both extant data and new data collection protocols.

To test the efficacy of different bad channel detection functions and determine the optimal criterion values for detection, we compared a series of automated options to a set of expert-identified bad channels for twenty files from an EGI dataset (each file had the same subset of 39 channels evaluated). These channels were determined by three expert reviewers (two external to the authors, all three with over 8 years each of EEG processing expertise in developmental and adult data). These 20 files were selected from a larger set because unanimous agreement was achieved across reviewers on the identity of good/bad channels (i.e. files in this test dataset had 100% expert reviewer agreement) to set clear benchmarks for the automated bad channel detection parameter testing. Expert review of bad channels was conducted prior to any automated testing to avoid bias. The files were then run through the HAPPE 1.0 legacy detection method for bad channels as well as several iterations of the Clean Rawdata function and combinations of Clean Rawdata with spectrum evaluation to optimize channel classification (shown in Table 2). Note that for iterations of Clean Rawdata with Flatline Criterion included, the Flatline default of 5 s was determined to be sufficient for detecting flat channels and was not manipulated further. We evaluated the outputs from each criterion for bad channel detection relative to the manually selected channels by summing the number of false negatives and false positives for each file and calculating the overall accuracy rate across files for that set of automated parameters. False negatives refer to channels that were manually marked as bad but not flagged as bad by the pipeline. False positives refer to channels that were manually marked as good but were marked bad by the pipeline. An extra emphasis was placed on finding the settings with high accuracy that produced the lowest number of false positives to avoid removing usable channels in the dataset. HAPPE+ER’s optimal settings produced 10 false negative channels and 9 false positive channels out of 780 channels across all 20 files for an overall accuracy rate of 97.6%.

### 2.7 Wavelet thresholding

To reduce the number of artifact-laden trials that must be rejected from any given file, artifact correction approaches may be applied to continuous EEG and ERP data prior to segmentation. Two dominant classes of artifact-correction approaches include independent component analysis (ICA) and wavelet-thresholding (used by HAPPE+ER). Briefly, ICA clusters data across electrodes into independent components that can segregate artifact from neural timeseries, while wavelet-thresholding parses data within frequency ranges using coefficients that can detect time-localized artifact fluctuations in either electrode data or independent components (see Gabard-Durnam et al., 2018 for detailed explanation). ICA requires rejection of entire timeseries which depends on sufficient segregation of neural from artifact data and appropriate selection of components for rejection to minimize the neural data rejected from artifact-laden timeseries. Wavelet-thresholding offers both time- and frequency-localized artifact detection and removal without distortion of the artifact-free underlying signal. That is, artifacts contaminating specific frequencies can be removed within the time-frame they occur without disturbing the remaining data in time or frequency dimensions with wavelet-thresholding. HAPPE+ER implements wavelet-thresholding to perform this artifact correction prior to segmentation and trial rejection. We evaluated a number of artifact correction strategies to optimize HAPPE+ER (detailed in the validation section that follows) and determined that wavelet-thresholding performed best in removing artifact and retaining neural (or simulated neural) signal. Several features of the wavelet thresholding step required optimization for ERP analyses in both adult and developmental populations (optimization analyses reported in Supplemental File 1). Note that the wavelet-thresholding step in HAPPE+ER should complete on the order of seconds for most user computing configurations and file lengths (though we have only recorded run-times for files up to 5 min length routinely, run-time has not scaled up with longer files). Users accustomed to grabbing coffee or lunch during ICA run-time will need to adjust schedules accordingly.

The user has two wavelet-thresholding options for ERP analyses within HAPPE+ER, specifically a “soft” or “hard” threshold to apply in removing artifacts from the signal. The soft threshold option may be optimal for users with minimally-artifact laden data (e.g., healthy adult samples) as this option can preserve ERP amplitudes best under conditions of generally clean signal. The hard wavelet threshold may be preferred by users with high- or variable-artifact contamination levels in their data (e.g., most developmental samples) as this option provides more rigorous artifact removal in conditions of high-artifact at the smallest cost to amplitude and preserves significantly more trials in artifact-contaminated data (see below section on HAPPE+ER comparison to other artifact-correction approaches section for simulated ERP results illustrating this threshold choice).

### 2.8 Filter for ERP using cutoffs

HAPPE+ER allows the user to choose both filter type and filtering frequencies for restricting data to frequencies of interest in ERPs (e.g. 0.1–30 Hz). Specifically, HAPPE+ER offers two filter types to choose from, (1) a Hamming windowed sinc FIR filter (EEGLAB’s pop_eegfiltnew function, filter order is estimated from user input frequency cutoffs), and (2) an IIR butterworth filter (ERPLab’s pop_basicfilter, order estimated as 3 * integer portion of (sampling rate /high-pass frequency)). Independent of filter type, users may input whatever high- and low-pass frequency cutoffs are desired.

### 2.9 Segmentation (recommended)

HAPPE+ER includes an optional data segmentation step for ERP analyses in which data is segmented around the events using the stimulus onset tags specified by the user. HAPPE+ER inherently corrects for any timing offset as part of the segmentation process, using the offset input by the user at the start of the HAPPE+ER run (if no timing offset is present, user may specify a zero-millisecond offset during that process). Two additional options are available for ERPs if segmentation is selected: baseline correction (recommended) and data interpolation within segments (optional artifact correction step), described in detail below. Users may segment their data regardless of whether these other options are enabled (see Fig. 1 for complete diagram of optional segment-related options in HAPPE+ER).

### 2.10 Baseline correction (recommended)

Users may perform baseline correction on segmented ERP data to correct drift-related differences between data segments. HAPPE+ER uses a standard mean subtraction method to achieve this correction where the mean value from the user-specified baseline window (defined relative to the stimulus onset) is subtracted from the data in the post-stimulus window. This step is implemented via the rmbase function in EEGLAB. The baseline correction option may be especially useful for users with saline-based acquisition systems and/or longer recording periods where drift is more commonly observed.

### 2.11 Bad data interpolation (optional)

For datasets (especially high-density datasets) where segment rejection would lead to an unacceptably low remaining number of segments for ERP analysis, users may choose an optional post-segmentation step involving the interpolation of data within individual segments for channels determined to be artifact-contaminated during that segment, as implemented by FASTER software (Nolan et al., 2010). Users should consult Nolan and colleagues (2010) for justification and further explanation of this approach should they choose this option. Each channel in each segment is evaluated on the four FASTER criteria (variance, median gradient, amplitude range, and deviation from mean amplitude), and the Z score for each channel in that segment is generated for each of the four metrics. Any channels with one or more Z scores that are greater than 3 standard deviations from the mean for an individual segment are marked bad for only that segment. These criteria may identify segments with residual artifacts in specific channels. Subsequently, for each segment, the channels flagged as bad in that segment have their data interpolated with spherical splines, as in FASTER. This allows users to maintain the maximum number of available segments, while still maximizing artifact rejection within individual segments. However, we caution users from implementing this option in cases where they have selected channel subset such that the channels are distributed with significant distance between them as the interpolation process would pull data from distal channels that does not reflect the appropriate activity profile for that scalp space.

### 2.12 Segment rejection (recommended)

For pre-segmented data or data run through HAPPE+ER’s optional segmentation step, users can choose to reject segments. If selected, segments can be rejected based on amplitude, joint probability, or both criteria. Amplitude-based rejection is useful for removing residual high-amplitude artifacts (e.g., eye blinks, drift from drying electrodes, discontinuities). If selected, users specify an artifact amplitude threshold such that any segment with at least one channel whose amplitude crosses the provided threshold will be marked for rejection. HAPPE+ER suggests an artifact threshold of − 200–200 for infant data, and − 150–150 for child, adolescent, and adult data. However, users are strongly encouraged to run the semi-automated HAPPE+ER setting on at least several files in their dataset to visually check that the selected amplitude results in appropriate segment rejection in their own datasets. Joint probability-based rejection catches other classes of artifacts, especially high-frequency artifacts like muscle artifact. Two joint probabilities are calculated with EEGLAB's pop_jointprob function. The joint probability of an electrode's activity in a segment given that same electrode's activity in all other segments is calculated (single electrode probability), and the joint probability of an electrode's activity in a segment given all other electrodes' activities for that same segment is calculated (electrode group probability). These joint probabilities are evaluated such that any segment is marked for rejection when either (1) a channel's single electrode probability or (2) its electrode group probability is outside of 3 standard deviations from the mean (setting performed well with semi-automated visual review). All segments marked from the user-selected steps are then rejected simultaneously in a single step. Notably, this segment rejection step may be run on all user-specified channels, or on a subset of channels for a specific region of interest (ROI). The ROI-channel subset option allows users to tailor segment rejection for a specific ROI analysis and potentially retain more data per individual if that ROI is less artifact-contaminated relative to other ROIs in the channels selected for HAPPE+ER processing.

### 2.13 Bad channel interpolation

For all HAPPE+ER runs, regardless of segmentation options, any channels removed during the bad channel rejection processing step are now subject to spherical interpolation (with Legendre polynomials up to the 7th order) of their signal. Channel interpolation repopulates data for the complete channel set specified by the user and reduces bias in re-referencing if the average re-reference option is selected. The identity of all interpolated channels, if any, for a file are recorded in HAPPE's processing report for users who wish to monitor the percentage or identity of interpolated channels in their datasets before further analysis.

### 2.14 Re-referencing (average or COI) (optional)

HAPPE+ER offers users the choice to re-reference the EEG data. If re-referencing, the user may specify either re-referencing using an average across all channels (i.e., average re-reference) or using a channel subset of one or multiple channels. For both re-referencing options, only channels within the user-specified channel subset selected for HAPPE+ER processing can be used for re-referencing. Re-referencing also reduces artifact signals that exist consistently across electrodes, including residual line-noise. During re-referencing, if there is a prior reference channel (e.g., an online reference channel), that channel’s data is recovered and included in the re-referenced dataset. For example, EGI data is typically online-referenced to channel CZ. In this example, users could now recover data at channel CZ by re-referencing to any other channel or channels in this step.

### 2.15 Split data by event markers

If the user enters more than one type of event marker (e.g. multiple conditions within a task, or multiple tasks that each contain one event marker), HAPPE+ER will split the data into files containing only the same event marker (e.g. condition-specific files) to facilitate further processing by event marker type, or to let the user trial-match across task conditions before generating ERP waveforms. HAPPE also allows for multiple markers to be categorized as a single condition, which will split the data into files containing only the event markers in that condition. For example, “happy_face” and “sad_face” event markers could be grouped into a “face” condition that would contain all trials for both event markers. Note that HAPPE+ER also retains the file that contains all event markers together. Thus, for files with multiple event markers read into HAPPE+ER, the data at this stage is parsed to provide: 1. File that contains all event markers, 2. files that each contain only trials with the same event-marker, and 3. files that contain only trials with the same condition.

## 3 HAPPE+ER outputs: pre-processed ERP data

HAPPE+ER generates several folders containing EEG files that are located within the user-specified folder of files for processing. EEG files are saved out after several intermediate processing steps so that users can explore in-depth and visualize how those steps affected the EEG signal in their own datasets. The intermediate files are separated into folders based on the level of processing performed on the data and include: (1) data after filtering to 100 Hz and line-noise reduction, (2) data post-bad channel rejection, (3) post-wavelet-thresholded data, and (4) data filtered at the user-specified ERP cutoffs. If segmenting is enabled, HAPPE+ER outputs one to three additional intermediate files: (5) post-segmented EEG data (always), (6) baseline-corrected data (if baseline correction is enabled), and (7) interpolated data (if bad data interpolation is enabled). If segment rejection is selected, HAPPE+ER saves the data post-segment rejection as well. All files at this stage contain data for individual trials (no trial averaging is performed during pre-processing).

HAPPE+ER outputs fully pre-processed files that are suitable inputs for further analyses in one of several formats, selected by the user at the start of the HAPPE+ER run, to increase compatibility with other software for data visualizations or statistical analyses. Options include.mat,.set, and.txt formats. We recommend using the.txt file format, which outputs three files in total: (1) A.txt file containing the average value across trials for each electrode at each sampling timepoint, (2) A.txt file containing the data for each electrode for each individual trial, and (3) An EEGLAB.set file of the fully processed EEG. For data with multiple event-markers, outputs for both the file containing all event markers and files that each contain a single event-marker are provided and labeled accordingly.

Finally, if the users ran HAPPE+ER in the semi-automated setting, the software generates an image for each file containing the fully processed data's power spectrum.

## 4 HAPPE+ER outputs: quality assessments

Alongside the fully processed data, HAPPE+ER also outputs the HAPPE Data Quality Assessment Report and the HAPPE Pipeline Quality Assessment Report, each described in detail below, for the file batch. Please note that the following list of data and pipeline quality metrics are current as of the resubmission of this manuscript (with HAPPE v2.1), but may be incomplete relative to subsequent versions of HAPPE+ER. Readers are encouraged to check the HAPPE user guide that comes with the software download for the complete and updated metric list and explanation for each HAPPE software version.

### 4.1 HAPPE data quality assessment report

HAPPE generates a report table of descriptive statistics and data metrics for each EEG file in the batch in a single spreadsheet to aid in quickly and effectively evaluating data quality across participants within or across studies. The report table with all these metrics is provided as a.csv file in the “quality_assessment_outputs” folder generated during HAPPE+ER. We describe each of these metrics below to facilitate their use to determine and report data quality.

#### 4.1.1 File length in seconds

HAPPE+ER outputs the length, in seconds, of each file prior to processing.

#### 4.1.2 Number of segments before segment rejection and number of segments post segment rejection

HAPPE+ER reports the number of segments before segment rejection and post segment rejection. If segment rejection is not enabled, these numbers are identical. If the user enabled segment rejection in HAPPE+ER, they may evaluate the number of data segments remaining post-rejection for each file to identify any files that cannot contribute enough clean data to be included in further analyses (user discretion based on study design and ERP of interest). The user may also easily tabulate the descriptive statistics for remaining segments to report in their manuscript's Methods section (e.g., the mean and standard deviation of the number of usable data segments per file in their study).

#### 4.1.3 Percent good channels selected and interpolated channel IDs

The percentage of channels contributing un-interpolated data (“good channels”) and the identity of interpolated channels are provided. Users wishing to limit the amount of interpolated data in further analyses can easily identify files for removal using these two metrics.

#### 4.1.4 % Variance retained

The percentage of the data retained post-wavelet-thresholding relative to pre-wavelet-thresholding is provided for each file. Users may wish to exclude participants from further analysis if they do not have sufficient data retained after artifact correction. A note for developmental data that large head movements or signal discontinuities in the data can result in very low values for this metric but do not indicate poor-quality data. Studies with infants where this is most common (e.g. poor head control, more movement in general) should rely instead on the correlation coefficients pre/post wavelet thresholding at specific frequencies that are produced in the pipeline quality report as described below.

#### 4.1.5 ICA-related metrics

As HAPPE+ER does not perform ICA on ERP data at time of publication, the metrics measuring ICA performance in HAPPE 2.2 for ERP analyses are assigned “NA.”

#### 4.1.6 Channels interpolated for each segment

If the user selected the Data Interpolation within Segments option of the additional segmenting options, HAPPE+ER will output a list of segments and the channels interpolated within each segment for each file. Otherwise, it will output “N/A.” Users wishing to limit the amount of interpolated data in further analyses can easily identify files for removal using this metric.

### 4.2 HAPPE pipeline quality assessment report

For each run, HAPPE+ER additionally generates a report table of descriptive statistics and data metrics for each EEG file in the batch in a single spreadsheet to aid in quickly and effectively evaluating how well the pipeline performed across participants within or across studies. Note that these metrics may also be reported in manuscript methods sections as indicators of how data manipulations changed the signal during preprocessing. The report table with all these metrics is provided as a.csv file in the “quality_assessment_outputs” folder generated during HAPPE+ER processing.

#### 4.2.1 r pre/post linenoise removal

HAPPE+ER automatically outputs cross-correlation values at and near the specified line noise frequency (correlation between data at each frequency before and after line noise removal) and at any additional specified frequencies. These cross-correlation values can be used to evaluate the performance of line noise removal, as the correlation pre- and post-line noise removal should be lower at the specified frequency, but not at the surrounding frequencies beyond 1–2 Hz. HAPPE+ER will automatically adjust which surrounding frequencies are reported depending on the user-identified line noise frequency. This metric can also be used to detect changes in how much line noise is present during the recordings (e.g. if generally cross-correlation values are high when study protocol is followed, indicating low line-noise removal from the data, but a staff member forgets to remove their cell phone from the recording booth for several testing sessions, the degree of line noise removal for those files summarized by this metric could be used as a flag to check in on site compliance with acquisition protocols).

#### 4.2.2 r pre/post wav-threshold

HAPPE+ER automatically outputs the cross-correlation values before and after wavelet thresholding across all frequencies and specifically at 0.5 Hz, 1 Hz, 2 Hz, 5 Hz, 8 Hz, 12 Hz, 20 Hz, 30 Hz, 45 Hz, and 70 Hz. These specific frequencies were selected to cover all canonical frequency bands across the lifespan from delta through high-gamma as well as the low-frequencies retained in ERP analyses. These cross-correlation values can be used to evaluate the performance of waveleting on the data for each file. For example, if cross-correlation values are below 0.1 for all participants in the sample, the wavelet thresholding step has not progressed as intended (users are advised to first check their sampling rate in this case and visualize several raw data files). Note that this measure may also be used to exclude individual files from further analysis based on dramatic signal change during waveleting (indicating high degree of artifact), for example if the 0.5 Hz or all-data cross-correlations are below some threshold set by the user (e.g., 3 standard deviations from the median or mean, r values below 0.1). For infant ERP data, these cross-correlation values are preferred for indicating which files should be removed for poor quality data over the % variance retention metric described above.

Through these quality assessment reports, HAPPE+ER aims to provide a rich, quantifiable, yet easily accessible way to effectively evaluate data quality for even very large datasets in the context of automated processing. Visual examination of each file is not required, although it is available. Over and above the purposes of rejecting files that no longer meet quality standards for a study and evaluating HAPPE+ER performance on a given dataset, we also hope to encourage more rigorous reporting of data quality metrics in manuscripts by providing these outputs already tabulated and easily transformed into descriptive statistics for inclusion in reports. Users may also wish to include one or several of these metrics as continuous nuisance covariates in statistical analyses to better account for differences in data quality between files or verify whether there are statistically significant differences in data quality post-processing between study groups of interest.

Several metrics may also be useful in evaluating study progress remotely to efficiently track the integrity of the system and data collection protocols. For example, the r pre/post line noise removal metric may indicate environmental or protocol deviations that cause significant increases in line noise in the data, and the Percent Good Channels Selected and Interpolated Channel ID metrics can be used to track whether the cap is being applied and checked for signal quality prior to paradigms or whether a channel (or channels) is in need of repair. For example, if the T6 electrode starts consistently returning bad data for a specific cap, it may need to be examined for repair. For further guidance about using the processing report metrics to evaluate data, users may consult the User Guide distributed with HAPPE+ER software.

## 5 Creating ERPs and calculating ERP values with the generateERPs script

HAPPE+ER comes with an optional post-processing script in the add-ons/generate subfolder, called ‘generateERPs’, with the capability to generate ERP waveforms and perform a series of calculations on the resulting ERPs. This script is separate from the HAPPE+ER pipeline’s script to encourage users to check the quality of their data and HAPPE+ER’s performance prior to generating the ERP figures and measures. Any files that do not pass data quality thresholds should be removed from the outputs folder prior to running the generateERPs script, otherwise they will be included in the subsequent figures and metrics. Much like HAPPE+ER, generateERPs runs on input taken directly from the command line and enables the saving and reloading of running parameters, continuing HAPPE+ER’s aim of making processing accessible to researchers of all levels of programming familiarity. The user must simply provide the full path of the processed folder created during the initial HAPPE+ER run and answer the prompts that follow.

To create ERP waveforms, users can select their channels of interest in an identical manner to channel selection in the HAPPE+ER pipeline (see above for more details). As part of channel selection, the user can additionally choose to include or exclude channels marked as “bad” during the HAPPE+ER processing run. If the user decides to exclude the bad channels detected during HAPPE+ER, they should first make a new.csv file that includes only the file names and bad channels columns from the Data Quality Assessment Report file generated by HAPPE+ER. This.csv file should be placed in the same folder as the processed data prior to running the generateERPs script. Note that if any files were removed post-processing due to insufficient data quality, they should be removed from the rows of this.csv as well.

**Fig. 2:**
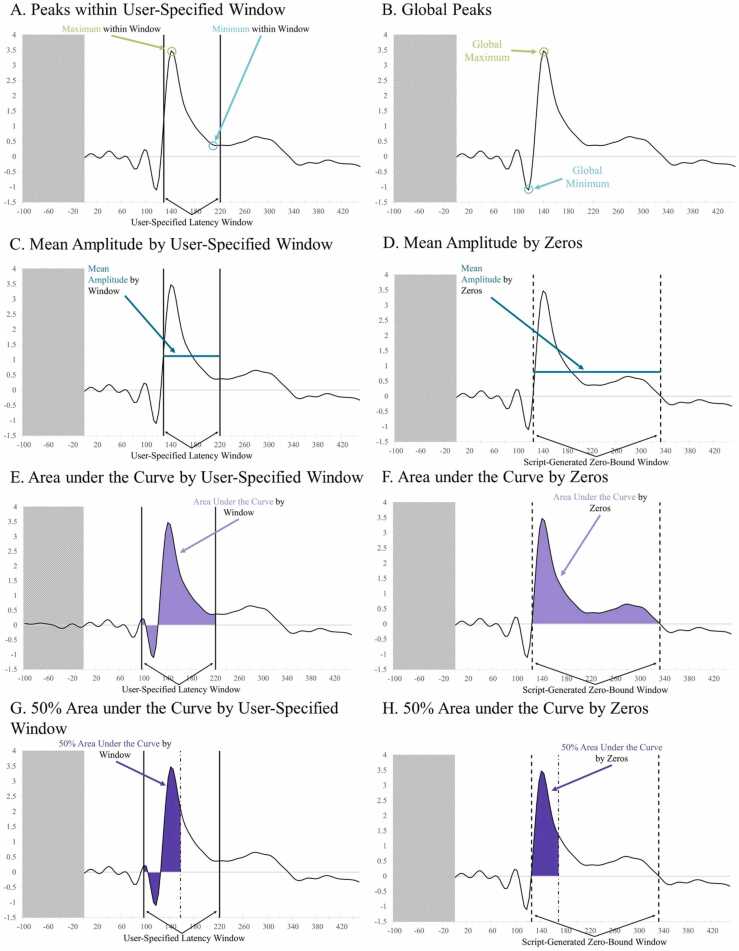
In all panels, the shaded area is not included in calculations. A) Circles the maximum (green) and minimum (blue) values within a specified latency window as indicated by vertical black lines. B) Circles the maximum (green) and minimum (blue) values across the entire ERP waveform. C) Mean amplitude within a specified latency window is represented by a teal horizontal line, with user-specified boundaries indicated by solid black vertical lines. D) Mean amplitude is represented by a teal horizontal line, and the boundaries created by the script at zero crossings in the data as dashed lines. E) Area under the curve is represented in light purple, with user-specified boundaries indicated by solid black vertical lines. F) Area under the curve is represented in light purple, and the boundaries created by the script at zero crossings in the data as dashed lines. G) 50% area under the curve is represented in dark purple. User-specified boundaries are indicated by solid black vertical lines. The alternating dot-dash line represents the latency at which 50% area under the curve is reached within the window. H) 50% area under the curve is represented in dark purple. Script-generated boundaries at zero-crossings are indicated by dashed black vertical lines. The alternating dot-dash line represents the latency at which 50% area under the curve is reached within the window.

The user is also asked whether they want to calculate a set of standard measures associated with the ERP for each file in the batch for subsequent statistical analysis. If so, the user must also specify: (1) latency windows of interest (e.g. 50–90 ms post-stimulus), (2) whether they anticipate a maximum or a minimum to be present in that window (i.e. a positive or negative ERP component, respectively), and (3) whether to calculate area under the curve and mean amplitude using temporal windows as bounds, using zero crossings present in the ERP data as bounds, or reporting measures with both methods (see Fig. 2).

The generateERPs script will create an ERP timeseries for each subject as well as an average ERP timeseries across subjects with both standard error and 95% confidence interval values for the average ERP, which are saved in a.csv output file in a new folder, “generated_ERPs”. The name of this file is “generatedERPs” plus any suffix associated with the selected data and the date. Three figures of these ERPs are also produced as generateERPs runs: (1) a figure containing the ERP of each subject, (2) the average ERP across subjects and the standard error, and (3) a combination of the first two figures. If enabled, generateERPs calculates the following values for each file and the average ERP across files, outputting them in an additional.csv file in the “generated_ERPs” folder.

### 5.1 Peak amplitudes and latencies

For each user-specified temporal window, generateERPs calculates the specified peak (either maximum or minimum depending on user input) and the latency at which it occurs (Fig. 2). The user may specify the same temporal window twice to request both a maximum and minimum within that window. Additionally, the global maximum and minimum of the timeseries (Fig. 2), a list of all maximums, a list of all minimums, and the latencies associated with each value are reported.

### 5.2 Mean amplitudes

If the user has selected mean amplitude based on windows, generateERPs calculates the mean amplitude of each user-specified latency window using said window’s start and end times as the upper and lower bounds.

If the user has selected to calculate mean amplitude based on zero crossings, generateERPs locates the zero crossings in the ERP and creates new latency windows using these crossing points, the starting latency, and the ending latency as bounds (Fig. 2).

### 5.3 Area under the curve

If the user has selected area under the curve based on windows, generateERPs calculates the area under the curve using the user-specified latency windows’ start and end times as the upper and lower bounds (Fig. 2). This method also reports the global area under the curve, calculated using absolute values, for the entire ERP waveform post-stimulus onset.

If the user has selected to calculate area under the curve based on zero crossings, generateERPs locates the zero crossings in the ERP and creates new latency windows using these crossing points, the starting latency, and the ending latency as bounds (Fig. 2).

### 5.4 50% Area under the curve and latencies

If the user has selected 50% area under the curve based on windows, generateERPs calculates 50% of area under the curve for each user-specified latency window for bounds and the latency at which it is reached (Fig. 2). This selection also reports the 50% area under the curve for the entire ERP waveform post-stimulus onset and its associated latency.

If the user has selected to calculate 50% area under the curve based on zeros, generateERPs creates latency windows using zero crossings in the ERP as bounds and reports the value and latency for this metric (Fig. 2).

We provide this script with the hopes of facilitating ERP visualization and analysis. Additionally, the speed, automation, and inherent compatibility with HAPPE+ER aligns directly with HAPPE+ER’s goals of providing an accessible and standardized method of examining EEG/ERP data.

## 6 HAPPE+ER comparisons to other pre-processing approaches

In this section of the manuscript, we compare HAPPE+ER’s artifact-correction methods to multiple other artifact-correction approaches (defined in detail below). These comparisons were useful in optimizing HAPPE+ER’s performance (all approaches were considered for inclusion in HAPPE+ER), and also provide empirical comparisons relevant to alternative published pipelines and common reported pre-processing strategies in the literature. We tested these approaches using both simulated ERP data and real developmental and adult ERP data (real ERP data shown in Supplemental File 4).

### 6.1 Artifact correction approaches defined

Wavelet-thresholding and independent component analysis (ICA) offer two separate strategies for artifact correction. We evaluated a number of contemporary algorithms using wavelet-thresholding or different automated algorithms to reject artifact ICs in ICA, namely: iMARA (Haresign et al., 2021), ICLabel (Pion-Tonachini et al., 2019), MARA (Winkler et al., 2011), Adjusted ADJUST (Leach et al., 2020), manual IC rejection for comparison, and an approach with no continuous data artifact rejection prior to segmentation. Thresholds for the automated algorithms were selected that best-performed for real developmental and adult data since the optimum algorithm for automated IC rejection with infant data was unclear, especially as multiple options have recently been released (e.g. Adjusted Adjust (Leach et al., 2020), iMARA (Haresign et al., 2021)). Therefore, the IC rejection step was first optimized for subsequent method comparisons (Supplemental File 2). Briefly, we note that automated component rejection performance, regardless of algorithm, depended highly on the rejection threshold selected. That is, there appears to be a tradeoff such that standard/default rejection thresholds (e.g. 0.5 artifact probability in MARA/iMARA) enable increased trial retention rates but remove a larger percent of the data and so result in more shrunken ERP amplitudes than the liberal rejection thresholds (Supplemental File 2). Without a clear optimal algorithm-threshold option, we proceeded to test here with the liberal iMARA 0.2 threshold and ICLabel 0.8 combinations for automated component rejection to preserve ERP amplitude as that is a key ERP measure of interest.

### 6.2 Comparison of artifact correction approaches for ERP analyses: simulated ERP data

We tested the performance of ICA and wavelet-based artifact-correction approaches using simulated ERPs with known temporal and amplitude properties embedded within real developmental baseline (resting-state) EEG data. This simulated ERP approach brings together the advantages of both simulated and real data: known signal properties of interest via simulated signal with the complexity and real artifact profiles of actual EEG recordings. Note that we piloted a completely simulated EEG dataset using brown and pink noise with the simulated VEP timeseries embedded in it and real artifact added to the data, but it proved to be not viable for testing. The difference in complexity between the simulated baseline data and the embedded artifacts from real EEG made artifact rejection trivial across methods and did not properly reflect the challenges of pre-processing real data.

Instead, our simulated ERP approach was performed as follows. The simulated VEP waveform was created using the SEREEGA EEGLAB plugin and referencing the VEP parameters set in the associated 2018 paper (Krol et al., 2018). We constructed the VEP from three ERP components (N1, P1, and N2) specifying the center of the peak, width, amplitude, and amplitude slopes as shown in Table 3. These parameters are the same as described in Krol et al. (2018), except we shifted the centers for each component forward in time by 100 msec to create a pre-ERP baseline period of 100 ms and enable baseline correction (e.g., the P1 was shifted from 100 msec from simulated data start to 200 msec post-data start so the first 100 msec could be used for a baseline later). For more on the rational for these settings, refer to Krol et al. (2018) and Pratt (2011).

**Table 3:** The parameters used to create each component of the simulated VEP, based on those set in Krol et al. (2018).

Component	Latency Centered Around (msec)	Width (msec)	Amplitude (μV)	Amplitude Slope
N1	170	60	-7.5	-2
P1	200	60	7.5	-2
N2	235	100	-10	-3

**Fig. 3:**
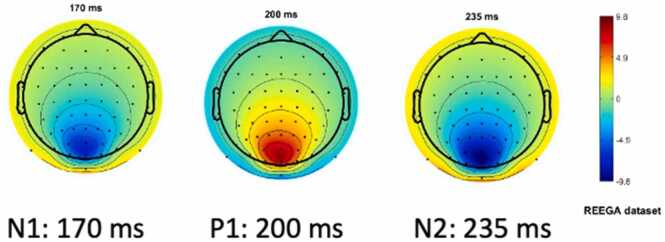
Topographical plots of each simulated VEP component (N1, P1, and N2) as generated by SEREEGA (Krol et al., 2018).

Once the components were defined, we generated a lead field using the New York Head field and the BioSemi 64 channel montage. All three VEP components were assigned to two sources bilaterally in the occipital lobe (Fig. 3). To the other sources we assigned a noise parameter created from pink noise with an amplitude of zero to create a flatline signal across the rest of the field. With these settings, we generated the simulated dataset with 60 epochs of 500 msec each for a total of 30 s of simulated data (60 simulated VEP trials).

After generating the simulated VEP signal, we prepared baseline (resting-state) EEG files from a developmental sample (ages 2–36 months) so that the ERP could be added to this data and submitted to artifact correction approaches for comparison. We generated two sets of simulated ERP data for these comparisons, a clean vs. artifact-added dataset, and a dataset of full-length files.

The clean vs. artifact-added dataset was created as follows to facilitate comparisons of performance on data with different artifact levels. We created sixteen 30-second files using continuous segments of relatively artifact-free (clean) baseline data from full-length baseline files. Next, from 30-second sections of the same individuals’ EEG that were artifact-laden, we ran ICA and extracted artifact independent components (identified by an expert and labeled artifact by both ICLabel and MARA automated algorithms). We inserted the artifact ICs into that individual’s clean 30-second data segment to create an additional 16 artifact-added files. To better equate performance of ICA with wavelet-thresholding on the shorter file lengths, we selected a subset of 39 spatially-distributed channels in the baseline EEG files as described with real VEP comparisons (Supplemental File 3). We then selected a channel from the simulated dataset with a stereotyped and prominent simulated VEP waveform, in this case Oz, and added its timeseries to each channel of the clean and artifact-added files to create two VEP file sets with a known ERP morphology. This allowed us to compare artifact correction effects on the simulated ERP at different spatial locations that may have different artifact-contamination profiles.

A full-length dataset was created to complement the controlled artifact testing in the first approach above by examining artifact correction performance on files with typical lengths and ranges of artifact-contamination seen in developmental studies (mean file length: 258 s, range: 119.996–509.996 s). This dataset was created using the addSimERP.m add-on script described later in this manuscript. To insert the VEP into a full-length dataset, we again used simulated VEP channel Oz from the dataset described above along with a set of 20 baseline EEG files from a developmental dataset. For each file, the script added the VEP timeseries to each channel of the data, repeating the timeseries in full as many times as possible to the nearest minute. Excess timepoints in the baseline data were then trimmed from the end of the dataset. For example, a file 125 s in length had 120 s of the repeating VEP timeseries added with the remaining 5 s removed. As with the clean and artifact-added files, this allowed us to examine the effect of pre-processing on a known ERP signal in a dataset of realistic length.

We provide the simulated VEP signal as well as these clean and artifact-added datasets and full-length files containing the embedded simulated VEP as a public resource for future pipeline validation and comparisons via Zenodo (https://zenodo.org/record/5172962).

With this simulated VEP embedded in real EEG datasets, we first batch-processed the files through HAPPE+ER up to the wavelet-thresholding step. At this point in processing, we either ran wavelet-thresholding (soft threshold option for the primary clean vs. artifact-added analyses, soft and hard thresholds illustrated for the additional full-length dataset) or ran ICA in EEGLAB (extended Infomax algorithm) and compared the following artifact correction approaches: wavelet-thresholding, ICA with MARA 0.5 automated rejection threshold, ICA with ICLabel 0.8 automated rejection threshold, iMARA with 0.2 automated rejection threshold (equivalent to.8 probability of artifact IC for rejection), and ICA with manual IC rejection. Manual rejection of ICs was carried out via EEGLAB by an expert with over a decade of experience in manually pre-processing EEG and ERP data, including using ICA-based rejection methods. Single-expert rejection was used to align with typical (at least as reported) laboratory practices in data processing. Given this analysis was conducted in response to reviewer request, the expert performed manual rejection while the automated approaches were also being tested but was not the same individual performing the automated analyses and was blind to the identity of the ICs and number of ICs rejected by the automated algorithms. Although the primary purpose of developing HAPPE+ER was to arrive at an automated preprocessing solution without subjective decisions, we also included the manual IC rejection as many labs currently use this approach and it has been shown previously to boost ICA performance in developmental data (e.g. Desjardins et al., 2021). Following the different artifact correction approaches applied to the simulated data, these datasets were processed further, including filtering to frequencies between 0.1 Hz and 35 Hz, segmentation, and baseline correction using a 100 msec baseline window.

Simulated VEP timecourses and component peak amplitudes were then extracted using HAPPE+ER’s generateERPs processing script across all datasets as follows. The mean VEP timecourse averaged across trials was calculated for each channel and averaged over clusters of electrodes to create three ROIs: occipital (O1, O2, E71, E75, E76), mid-frontal (F3, 19, Fz, 4, F4), and right temporal (110, 109, T4, 102, 98) (Supplemental File 3 for ROI map). The peak amplitudes for the three VEP components, N1, P1, and N2 were then extracted from each ROI using the following user-defined windows in generateERPs given the ground truth simulated VEP latencies: N1 (150–190 ms), P1 (180–220 ms), N2 (215–255 ms). To correct for any differences in the N1 component’s peak amplitude that could drive subsequent differences in measuring the P1 and N2 peaks if absolute amplitudes were used, we then calculated peak-to-peak amplitude values for all components following the N1 by subtracting the component of interest from the prior component’s peak amplitude (i.e. P1 – N1 peak values, N2 – P1 peak values). These N1, N1-P1, and P1-N2 amplitude values for all three spatial ROIs were then subjected to statistical analyses across pre-processing approaches.

We compared artifact-correction approaches’ performance for both the clean vs. artifact-added dataset and the full-length file dataset for the three ROIs (occipital, frontal, and right temporal) by evaluating the following criteria:

1. Rates of participant rejection. All files included in analyses were determined by an expert to have at least some sufficiently clean and usable trials, thus rates of rejecting entire files (i.e. all data or all trials removed) reflected un-necessary data attrition.
2. ERP morphology distortion. Specifically, for the clean vs. artifact dataset, using trial-matched ERPs generated across approaches, we evaluated whether A) the amplitude (peak and peak-to-peak amplitude magnitudes statistically compared) varied across methods for artifact correction relative to the known simulated ERP morphology, both as an independent signal and when embedded within real baseline EEG without artifact correction. B) We also evaluated whether amplitudes post-correction differed as a function of artifact-contamination level pre-correction. Better performing methods for correction should minimize the difference between the post-corrected clean data ERP amplitudes and the post-corrected ERP amplitudes from the artifact-added data as these files share the same underlying clean EEG signal. That is to say, the better-performing methods should not change the amplitude of the simulated ERP as a function of original artifact-level. So we compared the mean differences between clean and artifact-added simulated ERP amplitudes post-artifact correction across approaches as well.
3. ERP amplitude error around the mean. Evaluating the standard error (SE) around the peak ERP component amplitude means across participants in the clean vs. artifact dataset reveals the consistency of the estimates of the simulated ground truth values. This comparison is facilitated by the simulated conditions that do not contain any standard error around the mean amplitude values and because the exact same ERP was embedded into everyone’s EEG. Thus, any SE different from 0 reflects some error introduced into the estimates of amplitude. Of course, embedding the simulated data into the real EEG introduces SE. Therefore, artifact correction approaches are evaluated both on A) the absolute level of SE around ERP component estimates and B) whether they could reduce SE relative to the raw EEG conditions, suggesting higher consistency in the post-processed data across individuals. For the full-length datasets, the 95% confidence intervals around the mean simulated ERP were compared visually across approaches. This information combined with the amplitude distortion criterion above would indicate which artifact-correction approach most consistently and accurately re-captured the simulated ground truth values across individuals.
4. Trial rejection (sensitivity to artifact). Artifact correction approaches that were relatively more insensitive to artifact in the data compared to other approaches would be revealed by higher rates of subsequent trial rejection during processing (as retained artifact would be detected by the trial rejection criteria). To compare segment rejection across approaches, we used a voltage threshold-based criteria (−150 and 150 mV). That is, the best performing option would retain the most trials.

#### 6.2.1 Clean vs. artifact-added dataset comparisons

##### 6.2.1.1 Participant rejection

All methods tested on both clean and artifact-added datasets retained 100% of the participants. That is, there was no erroneous sample attrition regardless of processing strategy. No best performer with this criterion.

##### 6.2.1.2 ERP morphology distortion

**Fig. 4:**
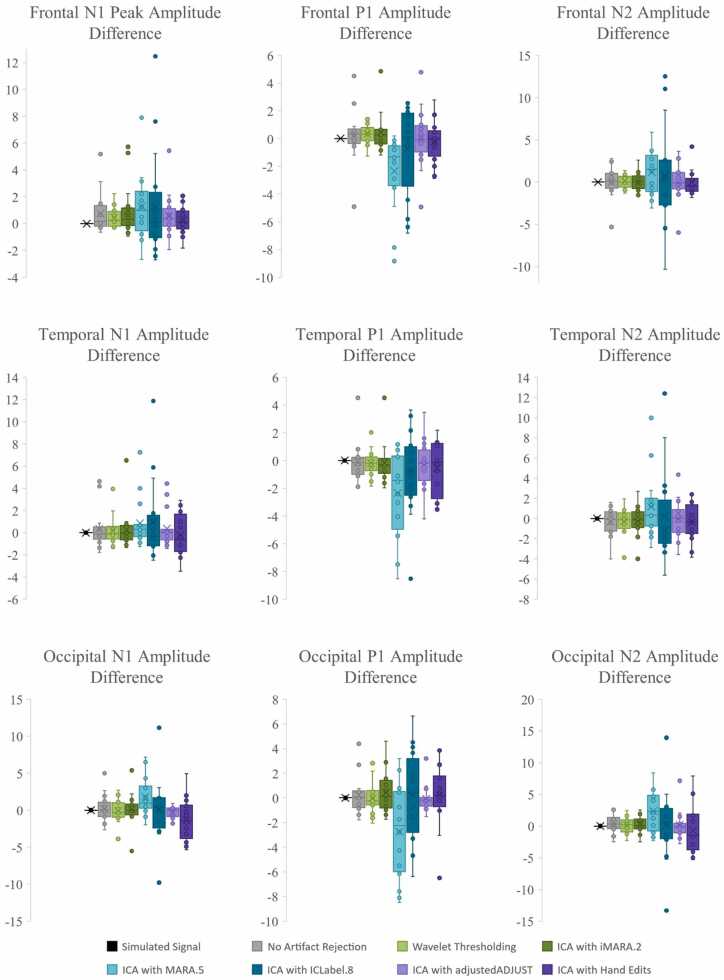
Box and whisker plots illustrating the difference in the post-artifact corrected amplitudes of components N1, P1, and N2 across the frontal, temporal, and occipital regions of interest as a function of pre-correction artifact level (i.e. clean ERP amplitude post-artifact correction minus artifact-added ERP amplitude post-artifact correction).

Next, approaches were compared with respect to effects on simulated ERP morphology across clean and artifact-added datasets for each spatial ROI with all trials (prior to bad trial rejection to evaluate the success of the artifact correction step specifically) (Table 4 and Fig. 4). Artifact-correction approaches were compared to the simulated ground truth values for the N1, N1-P1, and P1-N2 peaks and peak-peak amplitudes. Notably, while the simulated ERP was embedded in real baseline EEG data for the artifact-correction approaches to run, this embedding only minimally changed the peak simulated values across all ROIs in the clean dataset (ERP signal embedded in the clean real data produced peak amplitudes that were on average 99.68% the ground truth simulated value). Therefore, we proceeded with comparing approaches directly to the simulated ground truth given this negligible distortion to the simulated ground truth values in the clean real EEG.

**Table 4:** Statistics for the performance of various artifact rejection methods on simulated data ERP morphology amplitude.

	Simulated	Raw Data	Wavelet Thresholding	ICA with MARA.5	ICA with iMARA.2	ICA with ICLabel.8	ICA with Hand Rejection
Occipital							
Clean N1	-7.420	-7.514	**-7.180**	-6.038	-6.401	-5.804	-6.346
Clean N1-P1	16.030	15.864	**15.616**	13.372	13.400	12.241	14.111
Clean P1-N2	18.370	18.028	**17.718**	14.972	15.083	13.349	16.168
Mean amplitude difference from simulated		0.201	**0.435**	2.479	2.312	3.475	1.732
Artifact-Added N1	-7.420	-7.293	**-7.263**	-4.265	-6.292	-5.717	-7.678
Artifact-Added N1-P1	16.030	15.861	15.666	8.845	13.739	12.383	**15.683**
Artifact-Added P1-N2	18.370	17.913	**17.528**	9.838	15.230	13.416	17.087
Mean amplitude difference from simulated		0.251	**0.454**	6.291	2.186	3.435	0.629
Frontal							
Clean N1	-7.420	-7.407	**-7.238**	-5.540	-6.083	-6.387	-7.165
Clean N1-P1	16.030	15.689	**15.510**	12.462	12.847	13.151	14.983
Clean P1-N2	18.370	18.093	**17.845**	14.358	14.753	14.851	17.603
Mean amplitude difference from simulated		0.210	**0.409**	3.153	2.712	2.477	0.690
Artifact-Added N1	-7.420	-6.656	-6.773	-4.259	-5.136	-5.100	**-6.910**
Artifact-Added N1-P1	16.030	15.155	**15.351**	8.848	12.324	11.128	14.500
Artifact-Added P1-N2	18.370	18.316	**17.993**	10.266	14.976	13.179	17.259
Mean amplitude difference from simulated		0.564	**0.568**	6.149	3.128	4.138	1.050
Temporal							
Clean N1	-7.420	-7.662	**-7.544**	-5.420	-6.462	-6.569	-6.993
Clean N1-P1	16.030	15.641	**15.570**	12.168	13.062	13.435	14.502
Clean P1-N2	18.370	18.693	**18.619**	14.404	15.381	15.088	17.234
Mean amplitude difference from simulated		0.318	**0.278**	3.276	2.305	2.243	1.030
Artifact-Added N1	-7.420	-7.332	**-7.346**	-4.557	-6.178	-5.619	-7.227
Artifact-Added N1-P1	16.030	15.209	**15.193**	8.964	12.623	11.629	14.215
Artifact-Added P1-N2	18.370	18.913	**18.725**	10.882	15.478	14.042	17.051
Mean amplitude difference from simulated		0.484	**0.422**	5.806	2.514	3.510	1.109

Across both the clean and artifact-added datasets and all spatial ROIs (occipital, frontal, and right temporal), wavelet-thresholding returned the peak amplitude values closest to the simulated ground truth for the vast majority of ERP components (16 out of 18 components evaluated (9 of 9 clean components and 7 of 9 artifact-added components); mean difference from simulated amplitude value 0.4 mV, 3.1%) (Table 4). The ICA with manual rejection approach returned amplitude values closest to the simulated truth for the remaining two ERP components (frontal N1 in the artifact-added dataset, and occipital N1-P1 in the artifact-added dataset), but overall returned amplitudes on average 1.0 mV, 7.2% different from the simulated truth. The other ICA approaches returned amplitude estimates noticeably different from the simulated truth (ICA with MARA 0.5: mean 4.5 mV, 32.5% different; ICA with iMARA 0.2 mean 2.5 mV, 18.1% different; ICA with ICLabel 0.8 mean 3.2 mV, 22.6% different). That is, across VEP components, wavelet-thresholding returned amplitudes closest to the simulated truth, while no automated ICA approach performed nearly that well, and manual-rejection of ICs very rarely did. Still, within the ICA options, manual rejection did dramatically improve performance relative to even lenient automated rejection thresholds. Wavelet-thresholding provided the best performance in terms of minimal ERP morphology distortion across the scalp.

**Fig. 5:**
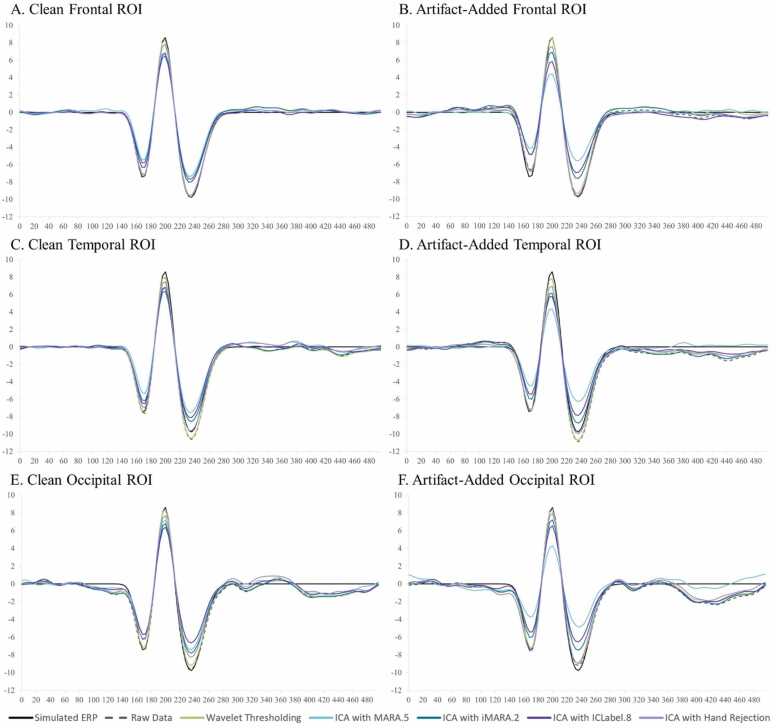
The resulting VEP waveform from the simulated VEP signal, data with the VEP signal added, and with a variety of artifact-reduction strategies. Excluding the simulated signal, all methods were processed using HAPPE’s line-noise reduction, filtering, segmentation, and baseline correction steps. The top row shows the waveform for clean (A) and artifact-added (B) data in the frontal ROI. The middle row shows the waveform for clean (C) and artifact-added (D) data in the temporal ROI. The bottom row shows the waveform for clean (E) and artifact-added (F) data in the occipital ROI.

Moreover, wavelet-thresholding returned estimates of the ERP peak amplitudes that were most similar across the clean and artifact-added conditions (Fig. 4, Fig. 5). That is, wavelet-thresholding in the artifact-added condition returned amplitudes that were extremely similar to the amplitudes found in the clean data (mean amplitude difference in artifact-added data amplitudes relative to clean data amplitudes: 0.1 mV, 1.0% difference) (Fig. 4). This difference between clean and artifact-added amplitude estimates for wavelet-thresholding was smaller than the difference observed between the raw EEG datasets (artifact-added vs. clean mean difference 0.2 mV, 1.7% difference). However, the ICA approaches variably reduced the ERP amplitudes more in the artifact-added condition compared to the clean condition (ICA with MARA 0.5 condition difference: 3.1 mV, 21% difference; ICA with iMARA 0.2 difference: 0.25 mV, 2.5% difference; ICA with ICLabel.8 difference: 0.99 mV, 7.7% difference; ICA with manual rejection: 0.5 mV difference, 3.8% difference). That is, the degree of artifact in the data affected the resulting ERP component amplitudes estimated post-artifact correction. This was observed even with the automated IC rejection algorithms set to liberal rejection thresholds, but was especially pronounced with the automated rejection algorithm set to the default threshold (21% amplitude estimate difference if the data corrected had 2 artifact ICs added to it). We also note that the artifact-added condition was by no means as artifact-heavy as many developmental EEGs the authors have observed in their time, thus this is likely a conservative estimate of these effects in the broader artifact conditions of most such studies. Given some degree of difference was observed here with manual IC rejection under conditions of some artifact, this difference may be notable even with expert IC rejection for datasets with more severe or variable artifacts present. This is a particularly concerning trend as variable degrees of artifact present would covertly influence the estimates of ERP parameters of interest in analyses.

Wavelet-thresholding may be spared from this artifact-dependent amplitude change because of how artifacts are rejected through ICA versus waveleting. That is, an entire file-length timeseries (IC) must be rejected during ICA, and more artifact will generally result in more timeseries rejected (which will shrink the signal amplitude if ICs are not extremely well segregated into artifact-only signal). However, wavelet-thresholding is both temporally and frequency-sensitive, and thus can remove artifacts within a timeseries within a specific frequency range without removing the entire timeseries for all frequencies. When tuned correctly, wavelet-thresholding is an extremely temporally- and frequency-sensitive approach to artifact removal (though not spatially sensitive to artifact clustering, as ICA is). These advantages may explain why wavelet-thresholding returned more consistent amplitudes regardless of artifact contamination. Alternatively, some may wonder if wavelet-thresholding is simply not removing any signal from the EEG and thus preserving amplitude (e.g. insensitive to artifact in the signal). Several indicators suggest otherwise. From this present analysis, the clean and artifact-added amplitudes produced by wavelet-thresholding were more consistent than in the raw conditions, indicating wavelet-thresholding was changing the artifact-added signal to better align it with the clean amplitudes (i.e. removing artifact that was distorting the amplitude). Further support for wavelet-thresholding removing artifact while retaining signal comes from the next two criteria evaluations on standard error across participants and rates of trial rejection post-artifact correction.

Wavelet-thresholding was the best-performing option for limiting ERP morphology distortion in the simulated ERP data across clean and artifact-added conditions in the three spatial ROIs tested.

##### 6.2.1.3 ERP standard error

Next, approaches were compared with respect to the standard error (SE) around the peak ERP component amplitudes across participants in the dataset to evaluate the consistency of the estimates of the simulated ground truth values (Table 5). This comparison is facilitated by the simulated conditions that do not contain any standard error around the mean amplitude values and because the exact same ERP was embedded into everyone’s EEG. Thus, any SE different from 0 reflects some error introduced into the estimates of amplitude. Of course, embedding the simulated data into the real EEG introduced SE, with higher SE estimates in the artifact-added dataset relative to the cleaner dataset across the three ROIs and ERP components (artifact SE mean: 0.527, clean SE mean: 0.449). As different artifact profiles were added to the different EEGs, smaller SE in the artifact-added conditions would also indicate more robust artifact-removal across artifact profiles. Artifact correction approaches were evaluated both on (1) the absolute level of SE around ERP component estimates and (2) whether they could reduce SE relative to the raw EEG conditions, suggesting higher consistency in the post-processed data across individuals. This information combined with the amplitude distortion comparison above would indicate which artifact-correction approach most consistently and accurately re-captured the simulated ground truth values across individuals.

**Table 5:** Statistics for the performance of various artifact rejection methods on simulated data ERP morphology standard error.

	Simulated	Raw Data	Wavelet Thresholding	ICA with MARA.5	ICA with iMARA.2	ICA with ICLabel.8	ICA with Hand Rejection
Occipital							
Clean N1	0.000	0.870	*0.84	1.033	0.977	1.223	0.984
Clean N1-P1	0.000	0.352	**0.419**	1.313	1.468	1.133	1.011
Clean P1-N2	0.000	0.547	**0.553**	1.366	1.472	1.205	1.043
Mean SE (clean)	0.000	0.590	**0.604**	1.237	1.306	1.187	1.013
Artifact-Added N1	0.000	0.944	*0.915	0.959	1.130	1.090	*0.846
Artifact-Added N1-P1	0.000	0.413	**0.459**	1.394	1.461	1.400	0.583
Artifact-Added P1-N2	0.000	0.596	*0.59	1.440	1.289	1.313	0.754
Mean SE (artifact-added)	0.000	0.651	**0.655**	1.264	1.293	1.268	0.728
Frontal							
Clean N1	0.000	0.527	*0.521	0.801	0.847	0.649	*0.468
Clean N1-P1	0.000	0.196	**0.207**	1.211	1.427	0.839	0.537
Clean P1-N2	0.000	0.259	*0.241	1.317	1.726	0.944	0.564
Mean SE (clean)	0.000	0.327	**0.323**	1.110	1.333	0.811	0.523
Artifact-Added N1	0.000	0.590	*0.504	0.773	1.021	1.026	*0.431
Artifact-Added N1-P1	0.000	0.423	*0.223	1.454	1.361	1.464	0.523
Artifact-Added P1-N2	0.000	0.247	**0.248**	1.688	1.692	1.499	0.562
Mean SE (artifact-added)	0.000	0.420	**0.325**	1.305	1.358	1.330	0.505
Temporal							
Clean N1	0.000	0.618	*0.581	0.759	0.819	0.760	0.720
Clean N1-P1	0.000	0.284	**0.285**	1.211	1.393	0.981	0.657
Clean P1-N2	0.000	0.390	*0.378	1.380	1.653	1.007	0.609
Mean SE (clean)	0.000	0.431	**0.415**	1.117	1.288	0.916	0.662
Artifact-Added N1	0.000	0.693	*0.655	*0.666	0.956	1.126	*0.606
Artifact-Added N1-P1	0.000	0.422	*0.41	1.113	1.478	1.474	0.623
Artifact-Added P1-N2	0.000	0.412	*0.37	1.242	1.588	1.481	0.652
Mean SE (artifact-added)	0.000	0.509	**0.478**	1.007	1.341	1.360	0.627

Across both the clean and artifact-added datasets and all spatial ROIs (occipital, frontal, and right temporal), wavelet-thresholding returned the lowest SE values for the vast majority of ERP components (14 out of 18 components evaluated (8 of 9 clean components and 6 of 9 artifact-added components); mean SE across components 0.467). Moreover, wavelet-thresholding reduced SE relative to the raw data for the majority of components (12 of 18 components evaluated, 7 of 9 artifact-added components, 5 of 9 clean components). That is, more frequent and larger reductions in SE occurred in the artifact-added condition (clean SE change: 0.002 reduction, artifact-added SE change: 0.041 reduction). Indeed, this differential SE reduction meant the wavelet-thresholded SE in the clean data was very similar to the wavelet-thresholded SE in the artifact-added data (second-smallest SE difference between conditions, with iMARA 0.2 showing the smallest difference between conditions (0.022)) but SE values much higher in both conditions than wavelet-thresholding (iMARA 0.2 SE overall mean 1.32). This pattern of results is consistent with successful artifact removal from the EEG data via wavelet-thresholding. The ICA with manual rejection approach returned the lowest SE values for the remaining four ERP components (occipital artifact-added N1, frontal clean and artifact-added N1, and temporal artifact-added N1), but returned higher mean SE values in both the clean and artifact-added conditions relative to both wavelet-thresholded SE values and the raw data SE values (e.g. manual rejection mean SE 0.283 larger than clean raw SE, and 0.093 larger than artifact-added raw SE). The automated ICA options all returned mean SE values over 1, suggesting less precise and more variable estimates of the ground truth ERP component amplitudes. In sum, only wavelet-thresholding successfully reduced standard errors around the ERP component amplitude estimates relative to raw data with artifact.

Wavelet-thresholding was the best-performing option overall for reducing error around the known simulated ERP signal regardless of artifact-levels before artifact correction.

##### 6.2.1.4 Segment retention

Finally, approaches were compared on their rates of segment retention as a benchmark for how much artifact remained after each artifact-correction approach that would require rejecting the entire trial during this pre-processing step. Although trial rejection is not a ground truth for presence or absence of artifact, it does facilitate comparisons between the approaches on the same trials, as relatively more trials retained using the same criteria should indicate cleaner underlying data compared to the other approaches. This criterion thus provides some information about which artifact-correction approaches were most sensitive to the artifact present in the data. We explored this question using a common voltage threshold for rejection (here, +/−150 mV in developmental EEG) (Table 6) for the clean EEG and artifact-added EEG datasets separately. A pattern of significant differences emerged when comparing these datasets using voltage thresholds for rejection (clean: F(5) = 8.549, p = 2 * 10^−6^, ${ƞ}_{p}^{2}$ = 0.363; artifact-added: F(5) = 23.509, p = 3.915 * 10^−14^, ${ƞ}_{p}^{2}$ = 0.610). Specifically, in the clean data, wavelet-thresholding, hand-rejection of ICs, and MARA0.5 all retained the highest number of trials (not significantly different from each other), and significantly more trials than iMARA0.2, ICLabel 0.8, and no artifact-correction prior to segment retention approaches (waveleting vs. iMARA p = 0.001, waveleting vs. ICLabel p = 0.010, waveleting vs. no correction p = 0.00045; manual rejection vs. iMARA p = 0.014, manual rejection vs. ICLabel p = 0.035, manual rejection vs. no correction p = 0.007; MARA vs. iMARA p = 0.012, MARA vs. ICLabel p = 0.017, MARA vs. no correction p = 0.007) In the artifact-added data, wavelet-thresholding, manual IC-rejection, and MARA 0.5 again retained the most trials (this time MARA0.5 retained the most, significantly more than wavelet-thresholding p = 0.003 but no other significant differences between these approaches), all significantly more than iMARA0.2, ICLabel0.8, and no artifact-correction approaches (MARA vs. iMARA p = 0.00009, MARA vs. ICLabel p = 0.0001, MARA vs. no correction p = 0.000008; wavelet vs. iMARA p = 0.0004, wavelet vs. ICLabel p = 0.003, wavelet vs. no correction p = 0.000007; manual rejection vs. iMARA p = 0.0003, manual rejection vs. ICLabel 0.0004, manual rejection vs. no correction 0.0003). The no artifact-correction approach retained significantly fewer trials than all artifact-correction approaches (p < 0.01 except iMARA p = 0.012), consistent with having the most rejectable artifact. We note that the MARA0.5 results should largely be ignored as this algorithm resulted in extreme amplitude reduction in the data, and thus more trials may have been retained simply because the entire signal was quite shrunken rather than because it was more free from artifact.

**Table 6:** Statistics for the performance of various artifact rejection methods on trial retention after segment rejection using HAPPE’s amplitude threshold criteria (−150 to 150 mV).

	Mean	Standard Deviation	Range	Mean	Standard Deviation	Range
Clean						
Raw Data	55.88	5.10	40–60	93.13	8.50	66.67–100.00
Wavelet Thresholding	59.13	2.39	51–60	98.54	3.98	85.00–100.00
ICA with MARA.5	59.75	0.58	58–60	99.58	0.96	96.67–100.00
ICA with iMARA.2	56.38	4.92	40–60	93.96	8.21	66.67–100.00
ICA with ICLabel.8	57.75	3.04	49–60	96.25	5.07	81.67–100.00
ICA with Hand Rejection	59.25	1.13	56–60	98.75	1.88	93.33–100.00
Artifact-Added						
Raw Data	48.19	7.12	31–57	80.31	11.87	51.67–95.00
Wavelet Thresholding	55.81	4.61	45–60	93.02	7.68	75.00–100.00
ICA with MARA.5	59.38	1.31	55–60	98.96	2.18	91.67–100.00
ICA with iMARA.2	50.06	7.51	32–100	83.44	12.51	53.33–100.00
ICA with ICLabel.8	51.00	6.60	32–58	85.00	11.01	53.33–96.67
ICA with Hand Rejection	58.25	1.61	54–60	97.08	2.69	90.00–100.00

Wavelet-thresholding tied as one of the most successful algorithms in terms of trial retention, and was the most successful automated algorithm that did not result in drastic amplitude reduction in the signal overall.

Across evaluation criteria for the clean vs. artifact-added dataset analyses, wavelet-thresholding consistently performed better than all other automated artifact-correction approaches, better than no artifact correction at all, and in most cases better than the manual IC rejection condition (with noted equivalency in trial retention) (Fig. 5).

### 6.2.2 Full-length dataset comparisons

Full-length EEG datafiles with simulated ERPs embedded were also examined to provide further comparison under conditions (more data samples per file and the full set of channels) where ICA might perform better than in the shorter segments required for above clean vs. artifact-added conditions. Here we also compared the soft and hard thresholding options for wavelet-thresholding to inform user selection.

#### 6.2.2.1 Participant rejection

All methods tested on full-length files retained 100% of the participants. That is, there was no erroneous sample attrition regardless of processing strategy. No best performer with this criterion.

#### 6.2.2.2 ERP morphology distortion

**Fig. 6:**
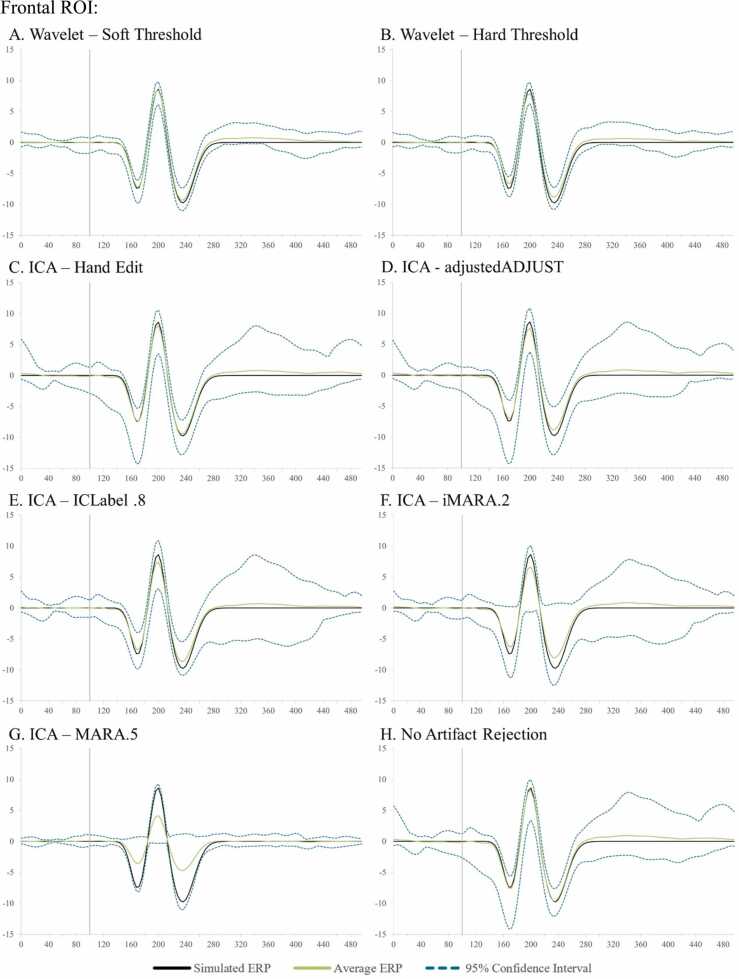
A series of plots comparing the simulated ERP signal (in black) with the average generated ERP (in green) for each artifact rejection method in the frontal region of interest. The 95% confidence intervals for the generated ERPs are illustrated by dotted teal lines.

**Fig. 7:**
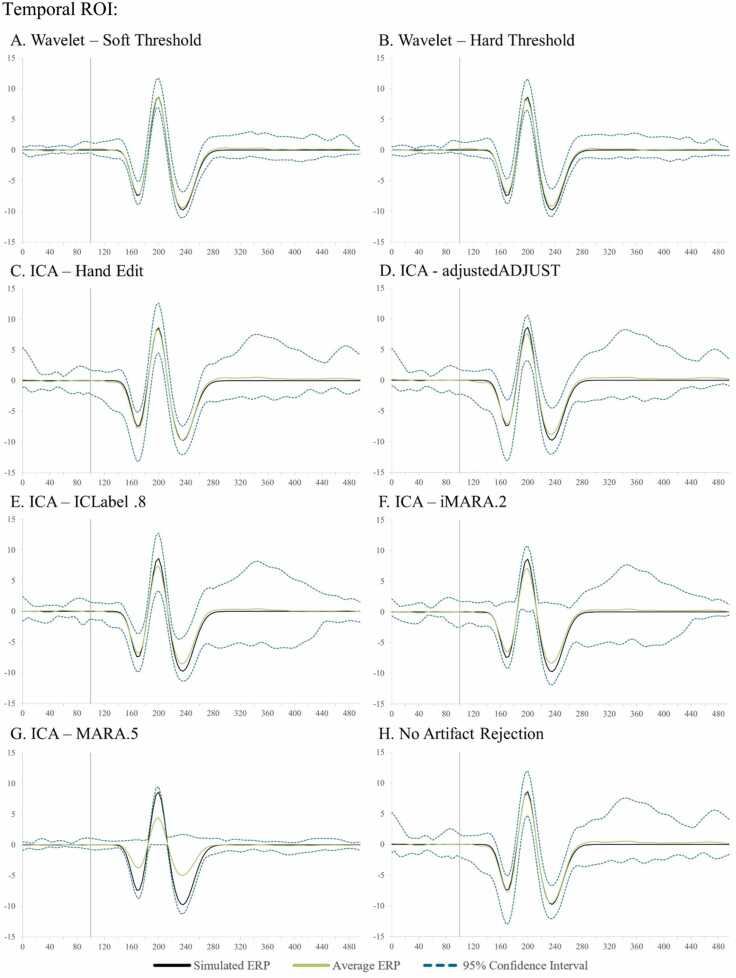
A series of plots comparing the simulated ERP signal (in black) with the average generated ERP (in green) for each artifact rejection method in the temporal region of interest. The 95% confidence intervals for the generated ERPs are illustrated by dotted teal lines.

**Fig. 8:**
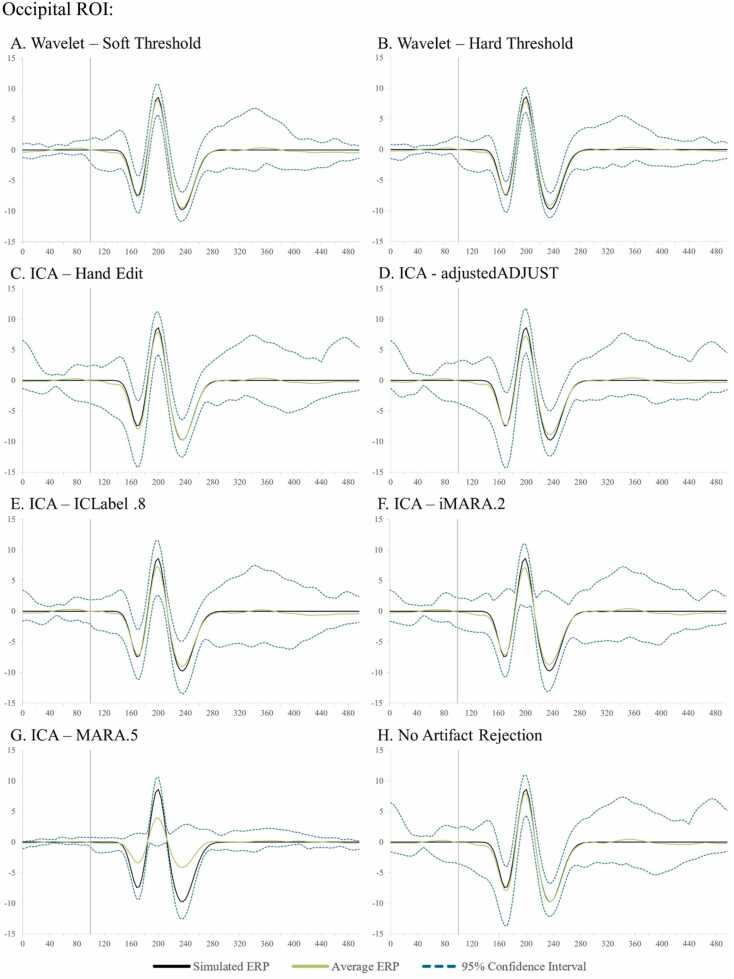
A series of plots comparing the simulated ERP signal (in black) with the average generated ERP (in green) for each artifact rejection method in the occipital region of interest. The 95% confidence intervals for the generated ERPs are illustrated by dotted teal lines.

The mean simulated ERP across files and 95% confidence intervals around the mean ERP are illustrated for the three different scalp ROIs in Fig. 6, Fig. 7, Fig. 8. Notably, the MARA0.5 algorithm for automated IC rejection significantly reduced ERP amplitude in the full-length data, and thus is not recommended in these conditions. Both wavelet-thresholding options performed best in terms of preserving amplitude while reducing the width of the 95% confidence interval relative to all IC options including manual rejection of ICs and the no-artifact correction option, regardless of the spatial ROI. The wavelet with hard threshold did result in slight amplitude loss relative to the soft threshold in these conditions, but also resulted in benefits with respect to segment retention rates outlined below.

Wavelet-thresholding with either soft or hard settings performed best in terms of reliably reproducing the simulated ERP signal across individuals and artifact-levels.

#### 6.2.2.3 Segment retention

Finally, approaches were compared on their rates of segment/trial retention as a benchmark for how much artifact remained after each artifact-correction approach that would require rejecting the entire trial during this pre-processing step. Once again, given MARA0.5 algorithm performance in terms of dramatic signal amplitude reduction, the MARA0.5 results are largely ignored here for the amplitude-based trial rejection test. A pattern of significant differences emerged when comparing these approaches using voltage thresholds for rejection (F(7) = 25.107, p = 1.39 * 10^−21^, ${ƞ}_{p}^{2}$ = 0.569). Specifically, as summarized in Table 7, aside from MARA0.5, wavelet-thresholding (hard threshold) and ICA with hand-rejection retained the most trials (not significantly different from each other p = 0.103; wavelet thresholding vs: Adjusted Adjust p = 0.002, ICLabel.8 p = 0.024, iMARA.2 p = 4.73 *10^−4^, no correction p = 1.17 *10^−7^; hand-rejection vs: Adjusted Adjust p = 3.88 *10^−8^, ICLabel.8 p = 1.17 *10^−4^, iMARA 1.1 *10^−5^, no correction p = 1.9 *10^−7^). Wavelet-thresholding with soft threshold retained significantly fewer trials than wavelet-thresholding with hard threshold (p = 1.1 *10^−7^) and ICA with manual IC rejection (p = 3.4 * 10^−5^), but was equivalent to all automated ICA options statistically (Adjusted Adjust p = 0.657, iMARA0.2 p = 0.406, ICLabel.8 p = 0.106) and retained significantly more trials than the raw condition of no artifact-correction (p = 4.93 *10^−7^). The no artifact-correction condition retained significantly fewer trials than all methods (all p < 0.001). This pattern of results provides further support for the use of artifact-correction approaches to improve data quality and data retention.

**Table 7:** Statistics for the performance of various artifact rejection methods on trial retention after segment rejection using HAPPE’s amplitude threshold criteria (−150 to 150 mV).

	Number of Segments Retained			Percent Segments Retained		
	Mean	Standard Deviation	Range	Mean	Standard Deviation	Range
Raw Data	254.55	134.52	92–625	50.24	15.38	30.67–85.00
Wavelet Thresholding, Soft	338.10	172.70	152–824	66.75	16.37	39.70–98.75
Wavelet Thresholding, Hard	412.80	199.42	201–960	81.26	14.63	49.55–99.58
ICA with adjustedADJUST	345.70	193.84	120–841	65.68	15.20	38.79–94.17
ICA with MARA.5	496.65	252.26	0–1013	92.50	23.50	0.00–100.00
ICA with iMARA.2	350.15	187.48	131–808	67.50	16.47	40.61–96.67
ICA with ICLabel.8	366.40	190.51	135–897	71.00	14.42	48.18–97.08
ICA with Hand Rejection	440.70	209.93	186–956	84.97	8.52	63.49–98.33

The pattern of results over the clean vs. artifact-added and full-length datasets suggests that wavelet-thresholding rejects more artifact and reproduces the simulated ERP signal more consistently across individuals than any other approach tested in this developmental EEG data. Though ICA, especially with manual IC rejection, may prove useful under other circumstances for ERP analyses, (e.g. perhaps long, clean adult EEG recordings), wavelet-thresholding as tested here in HAPPE+ER provides a flexible, accurate, and automated solution for artifact-correction across the lifespan.

## 7 HAPPE+ER comparisons to other automated pre-processing pipelines

Finally, we provide both conceptual and empirical comparisons to other automated pre-processing pipelines for ERP analyses. Specifically, we address the two recent pipelines also tested in ERP data, EEG-IP-L (Desjardins et al., 2021) and MADE (Debnath et al., 2020).

### 7.1 Conceptual differences between pipelines

There are multiple conceptual, goal, and user-related differences between available pipelines. The EEG-IP-L pipeline employs lossless methods to annotate datasets where artifact is present, and has recently been shown to outperform MADE in retaining trials and recovering ERP effects (Desjardins et al., 2021). EEG-IP-L offers a suite of useful tools and visualizations, especially for advanced users with access and knowledge of computing clusters (at time of press, cluster access was required for the publicly-available version of EEG-IP-L for researchers in the USA). EEG-IP-L uses the ICLabel algorithm we tested in the current manuscript, although we tested more lenient automated criteria and manual IC rejection in the absence of EEG-IP-L’s prior annotation practices and multiple ICA decompositions. EEG-IP-L authors recommend manual inspection and editing of ICA for optimal performance (a recommendation we agree with based on how automated IC rejection has performed in our independent comparisons). In this way, EEG-IP-L serves both a different userbase and different pipeline goals from HAPPE+ER.

The MADE software offers a more directly comparable option that also aims for fully-automated pre-processing of ERP data. MADE is designed for developmental data only while HAPPE+ER is appropriate for both developmental and adult ERP data. Still, like HAPPE+ER, MADE offers the same set of pre-processing steps for ERP data, with several differences in implementation for specific steps described next. For bad channel detection, MADE uses FASTER’s criteria for detecting bad channels, which evaluates correlation relative to other channels, variance of a channel relative to other channels, and Hurst exponent for a channel. MADE also rejects channels with large amplitude changes and excessive EMG for more than 20% of the recording. HAPPE+ER includes correlation relative to other channels, and explicit flat-channel detection (which should be caught in FASTER), but further evaluates the power spectrum (which can catch EMG-contaminated channels amongst other artifacts that shift the power spectrum) and line noise contamination to detect further classes of bad channels (some of these may fail FASTER criteria or be caught for excessive EMG activity in MADE but are not all explicitly targeted).

Both MADE and HAPPE+ER pipelines also perform artifact correction. MADE employs ICA with automated IC rejection using Adjusted ADJUST (Leach et al., 2020), which searches for ocular artifact (blinks, EOG artifacts, saccades) and generic discontinuities in the EEG data. HAPPE+ER uses wavelet-thresholding, which can theoretically detect artifacts across a broader set of classes (e.g. EMG, heart-rate, respiration, ocular, discontinuities, movement, etc). Though we tested Adjusted Adjust for automated rejection and found wavelet-thresholding outperformed it on simulated ERP data above, this was outside of the context of MADE’s pipeline and may not reflect Adjusted ADJUST’s performance with MADE’s other pre-processing steps.

Pipelines also differ in how they perform bad segment rejection. MADE performs bad segment rejection by searching for and rejecting segments that surpass a voltage threshold over ocular regions first, then in greater than 10% non-ocular electrodes, and for segments determined to be bad for fewer than 10% of non-ocular electrodes, the data for those segments is interpolated using data from nearby channels. HAPPE+ER offers the user several approaches to handle bad segments, including optional interpolation (interpolation is not optional in MADE), and rejection via voltage threshold criteria, similarity criteria (via EEGLAB’s jointprob function) to both other segments for that electrode and other electrodes for that segment, or the combination of these two similarity and voltage criteria. Moreover, users may reject segments using all electrodes or evaluate an ERP’s ROI specifically to optimize segment retention for that analysis. HAPPE+ER offers a simple re-run function to evaluate multiple ROIs for segment rejection within the same dataset if ROI-based segment rejection is preferred.

Finally, MADE does not offer a complement to HAPPE+ER’s generateERP post-processing functionality for generating ERP metrics and figures, nor can it easily accommodate low-density EEG data for ERP analyses due to reliance on ICA for correction.

### 7.2 Empirical differences between pipelines

Given that HAPPE+ER and MADE pipeline goals are most closely aligned in striving for fully-automated ERP pre-processing for a broad userbase, we also empirically tested performance on developmental ERP data for HAPPE+ER (hard wavelet-threshold option) relative to MADE pipeline using the full-length simulated ERP dataset and then real ERP datasets from 4-month and 10-month infants. We used the version of MADE publicly-available code downloaded on December 20, 2021, and though we had to implement code changes to render the code functional, we do not believe these changes altered MADE’s functionality beyond what the authors intended. We processed the simulated ERP, 4-, and 10-month data through both MADE and HAPPE+ER after first excluding the rim channels for the data as MADE recommends this for optimal performance in their pipeline (channels excluded include ‘E17′ ‘E38′ ‘E43′ ‘E44′ ‘E48′ ‘E49′ ‘E113′ ‘E114′, ‘E119′ ‘E120′ ‘E121′ ‘E125′ ‘E126′ ‘E127′ ‘E128′ ‘E56′ ‘E63′ ‘E68′, ‘E73′ ‘E81′ ‘E88′ ‘E94′ ‘E99′ ‘E107′). As MADE only specifies voltage-related segment rejection, we used HAPPE+ER’s voltage criteria only for segment rejection (−150 and 150 mV for both HAPPE and MADE, following MADE’s recommendation for infant data). Pre-processed data from both pipelines were processed using HAPPE+ER’s generateERPs script as described previously to extract mean ERP timeseries, measures of error around the mean (standard error, confidence intervals), and peak amplitude values for the N1, P1, and N2 components to evaluate N1, N1-P1, and P1-N2 morphology.

We evaluated pipeline performance on the following criteria:

1. File retention rates of participant rejection. All files included in analyses with simulated and real ERP data were determined by an expert to have at least some sufficiently clean and usable trials, thus rates of rejecting entire files (i.e. all data or all trials removed) reflected un-necessary data attrition.
2. ERP morphology. Specifically, for simulated ERP data, we evaluated whether the simulated signal amplitude and timing were recapitulated data post-artifact correction, as well as the width of the 95% confidence intervals around the mean amplitude prior to segment rejection. Morphology comparisons were performed for the simulated ERP over frontal, temporal, and occipital regions of interest (ROIs) as before. Better pipeline performance would be indicated by closer approximation of the simulated signal amplitude and timing, and smaller 95% confidence intervals around the amplitude. The same analyses were done for the real ERP data, but these would simply indicate pipeline differences rather than indicate superior performance in the absence of a ground truth.
3. Segment retention (sensitivity to artifact). A pipeline that was relatively more insensitive to artifact in the data compared to the other would be revealed by higher rates of subsequent trial rejection during processing (as retained artifact would be detected by the trial rejection criteria). Here across both simulated and real ERP datasets, we used segment rejection voltage criteria (−150 to 150 mV) to evaluate which pipeline preserved more trials as the better-performing pipeline, noting that MADE’s criteria should reject fewer segments since it includes interpolation for some cases where HAPPE+ER would reject segments.

#### 7.2.1 Simulated ERP analyses

##### 7.2.1.1 File retention rates

HAPPE+ER retained 100% of the files and MADE retained 100% of the files with simulated ERPs embedded. No best performer using this criterion.

##### 7.2.1.2 ERP morphology and error

**Fig. 9:**
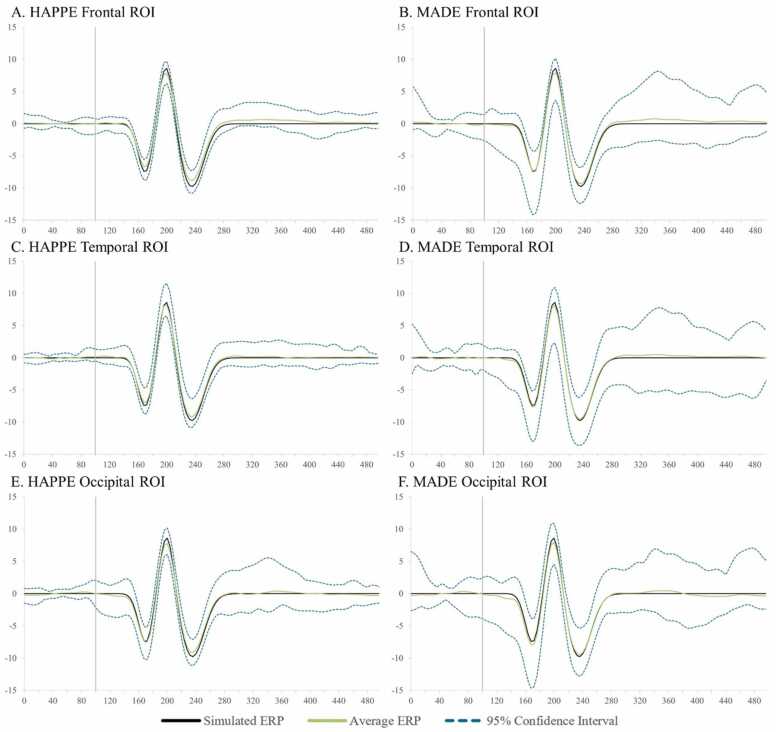
A series of plots comparing the simulated ERP signal (in black) with the average generated ERP (in green) between HAPPE and MADE across regions of interest. The 95% confidence intervals for the generated ERPs are illustrated by dotted teal lines.

Pipeline effects on the simulated ERP morphology across the three regions of interest (frontal, temporal, and occipital) are summarized visually in Fig. 9. Across the scalp, both pipelines returned accurate mean simulated ERP morphology with minimal distortion. HAPPE returned consistently narrower 95% confidence intervals around the mean simulated ERP, suggesting more consistent and accurate artifact correction performance across developmental files.

##### 7.2.1.3 Segment retention (sensitivity to artifact)

Pipelines were also compared on their rates of segment retention as a benchmark for how much artifact remained after each pipeline’s pre-processing steps including artifact-correction (Table 8). Although trial rejection is not a ground truth for presence or absence of artifact, it does facilitate comparisons between the pipelines on the same trials, as relatively more trials retained should indicate cleaner underlying data compared to the other pipeline. Here using the voltage-based criteria, we compared MADE’s retained segments with HAPPE+ER run on the same set of channels. HAPPE+ER retained significantly more trials relative to MADE (HAPPE+ER vs. MADE t(19) = 5.72, p = 1.6 * 10^−5^). That is, HAPPE performed better than MADE for the segment retention criteria.

**Table 8:** Statistics for the performance of HAPPE and MADE on trial retention after segment rejection.

	Number of Segments Retained			Percent Segments Retained		
	Mean	Standard Deviation	Range	Mean	Standard Deviation	Range
HAPPE	412.8	199.42	201–960	81.26	14.63	49.55–99.58
MADE	309.25	178.63	116–820	59.08	15.29	35.37–95.28

These comparisons with simulated ERPs embedded in real developmental EEG data provide support for the use of HAPPER+ER for robust developmental ERP pre-processing.

#### 7.2.2 Real developmental ERP analyses

For final comparisons, we also evaluated pipeline performance using real infant visual evoked potential data from 4- and 10-month old babies (details of paradigm and data collection provided in Supplemental File 2).

#### 7.2.2.1 File retention rates

While HAPPE+ER retained 100% of files, MADE rejected a small percentage of files known to have usable data in both the 4- and 10-month datasets, representing unnecessary data attrition (Table 9). Moreover, we evaluated how many files were effectively usable for analyses, here having at least 15 trials of retained VEP data post-processing, and using these criteria, both HAPPE+ER and MADE rejected files at 4-months, though MADE rejected far more files at this timepoint (MADE: 65% file retention vs. HAPPE+ER: 95% file retention rate in this condition). Moreover, MADE rejected files in this condition at 10-months of age while HAPPE+ER retained the full sample of usable participants (MADE: 88% file retention). Thus, MADE may induce some unnecessary file attrition in developmental ERP studies.

**Table 9:** Statistics for the performance of MADE versus HAPPE on file retention using amplitude threshold segment rejection criteria.

	MADE - MADE's Voltage Threshold		HAPPE - Voltage Threshold		HAPPE - Voltage Threshold on ROI	
	Percent Files Retained	Percent Files with at least 15 Trials	Percent Files Retained	Percent Files with at least 15 Trials	Percent Files Retained	Percent Files with at least 15 Trials
4-month data	95.65	65.22	100.00	95.83	100.00	100.00
10-month data	94.12	88.24	100.00	100.00	100.00	100.00

#### 7.2.2.2 ERP morphology

**Fig. 10:**
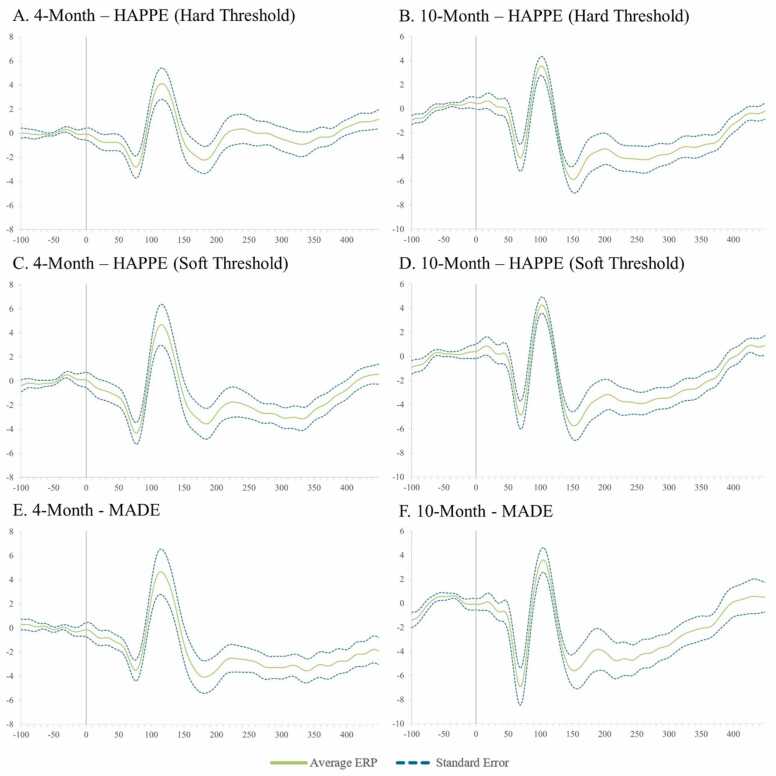
The generated VEP and standard error for 4-month data processed with HAPPE using hard (A) and soft (C) thresholds, 10-month data processed with HAPPE using hard (B) and soft (D) thresholds, 4-month data processed with MADE (E), and 10-month data processed with MADE (F). The standard error is represented by the dashed lines. In all images the files have been limited to those which MADE retained at least 15 segments and are segment matched (42 epochs for 4-month data, 41 epochs for 10-month data).

Next, pipelines were compared with respect to effects on VEP morphology across 4- and 10-month datasets using trial number matched datasets (matched to whichever pipeline returned fewer trials for each participant and including only participants where MADE and HAPPE+ER both retained at least 15 trials of VEP) (Fig. 10). Thus, in the subsample of files and trials, there were largely no differences in morphology or standard errors between pipelines under trial-matched conditions visually. Both the hard and soft wavelet thresholds are shown in the real developmental data for comparison. We note that differences in standard error between pipelines may be expected if the ERP morphology without trial-matching were performed given the trial retention results described below and the simulated ERP results above where all trials were compared.

#### 7.2.2.3 Segment retention

Finally, pipelines were compared on their rates of segment retention as a benchmark for how much artifact remained after each pipeline’s pre-processing steps including artifact-correction (Table 10). Here we compared MADE’s retained trials with HAPPE+ER run on the same set of channels, as well as HAPPE+ER run in the ROI segment rejection setting. Both HAPPE+ER settings retained more trials relative to MADE in both the 4-month and 10-month datasets (4 months: HAPPE+ER (all channel setting) vs. MADE t(22) = 7.67, p = 1.19 * 10^−7^, HAPPE+ER (ROI setting) vs. MADE t(22) = 13.60, p = 3.47 * 10^−12^; 10-months: HAPPE+ER (all channel setting) vs. MADE t(16) = 6.30, p = 1.1 * 10^−5^, HAPPE+ER (ROI setting) vs. MADE t(16) = 6.52, p = 0.7 * 10^−5^). HAPPE+ER’s ROI setting retained significantly more trials than HAPPE+ER evaluated over all channels at both ages as well (4 months: all channels vs. ROI t(22) = 8.84, p = 1.08 * 10^−8^, 10 months: all channels vs. ROI t(16) = 3.75, p = 0.002). Thus, for developmental researchers interested in ERPs that are already localized spatially, the HAPPE+ER ROI option for trial rejection may further improve the count of usable trials for analyses.

**Table 10:** Statistics for the performance of MADE versus HAPPE on trial retention using amplitude threshold segment rejection criteria.

	MADE - MADE's Voltage Threshold		HAPPE - Voltage Threshold		HAPPE - Voltage Threshold on ROI	
	Number of Segments Retained	Percent Segments Retained	Number of Segments Retained	Percent Segments Retained	Number of Segments Retained	Percent Segments Retained
4-month data						
Mean	29.78	27.14	*60.09	*56.23	*^ 93.17	*^ 89.44
Standard Deviation	30.05	23.18	36.59	25.31	30.86	18.33
Range	0–116	0.00–89.23	2–173	2.04–97.17	25–199	25.51–100.00
10-month data						
Mean	37.24	37.49	*83.71	*79.60	*^ 103.71	*^ 96.53
Standard Deviation	16.97	16.29	24.84	16.39	33.86	4.41
Range	0–64	0.00–62.38	44–145	34.92–100.00	56–218	87.07–100.00

In summary, across evaluation criteria in this infant VEP dataset, MADE resulted in file attrition relative to HAPPE+ER, and while both pipelines produced robust ERP morphology in trial-matched conditions, HAPPE+ER retained significantly more trials per file than MADE, regardless of HAPPE+ER rejection approach. These results may reflect HAPPE+ER’s sensitivity to more artifact classes in artifact correction or superior performance of wavelet thresholding relative to ICA more broadly as we have previously shown. These comparisons in developmental data provide support for the use of HAPPER+ER for robust developmental ERP pre-processing.

## 8 Validating HAPPE+ER and pipeline comparisons in user data

To support testing HAPPE+ER performance in the context of each user’s data, and to bolster the ability for scientists to test which pipeline of the many emerging options performs best for their data needs, we introduce the first of a new set of validation scripts in HAPPE that can be found in the add-ons\validate folder, addSimERP.m. This script easily enables the user to add the simulated ERP signal described above to their continuous baseline/resting-state EEG data as described and used in this manuscript. As with the main HAPPE script and generateERPs, the user can select either all the channels to add the ERP timeseries or a subset of channels using the include/exclude methods described previously. While currently only supporting continuous EEG files in.set format with channel locations included, the authors intend to enable the script’s functionality to include inputs in.mat and.raw formats as well. Similarly, addSimERP.m only allows a simulated VEP timeseries to be added to the provided data but this selection will be expanded to facilitate a choice of other simulated ERP timeseries and enable validation across a variety of waveforms.

## 9 Implementing HAPPE+ER

HAPPE+ER runs entirely through the MATLAB command line, collecting processing parameters without the user needing to navigate or alter the pipeline’s code. This reduces the chance of accidentally breaking the code, entering incorrect parameters for the desired analysis, and the need to have prior knowledge of coding or MATLAB, enabling users of wide range of backgrounds and levels of familiarity to easily run the pipeline. To run HAPPE+ER, simply open MATLAB, navigate to the HAPPE 2.0 folder, and open the HAPPE 2.0 script. In the “Editor” tab at the top of the screen, click “Run” and follow the prompts as they appear in the MATLAB command line. After entering all relevant inputs to the command line, HAPPE+ER will run automatically through completion.

HAPPE+ER code and user guide are freely available at: https://github.com/PINE-Lab/HAPPE. The data used in this manuscript are freely available via Zenodo at: https://zenodo.org/record/5172962.

## Funding

This work was supported by a grant from the 10.13039/100000865Bill & Melinda Gates Foundation, Grant number 598476 to LGD.

## Declaration of Competing Interest

The authors declare that they have no known competing financial interests or personal relationships that could have appeared to influence the work reported in this paper.

## Data Availability

I have shared links to the data and code within the manuscript.
