# Supplementary material for: The HAPPE plus Event-Related (HAPPE+ER) software: A standardized preprocessing pipeline for event-related potential analyses
Source: Dev Cogn Neurosci. 2022 Jul 19;57:101140. doi: 10.1016/j.dcn.2022.101140 (PMC9356149; doi:10.1016/j.dcn.2022.101140)
Supplement: Supplementary material [file mmc1.docx]

### *Supplemental File 1*

### *Optimization of wavelet thresholding resolution for ERP analysis*

Wavelet thresholding decomposes the data out into frequency ranges dictated by a resolution parameter, but the appropriate resolution level for ERP analyses has not been validated before. The resolution level is critical as it determines the bins of frequencies that are considered together for identifying and removing artifacts. Therefore, across adult and developmental datasets, we examined how wavelet resolution level influenced the VEP waveform, number of segments retained, and the degree of change in the data across frequencies (via quality control cross-correlation values) to optimize this approach for ERP analyses before testing approaches involving wavelet-thresholding. Specifically, we evaluated decomposing the data so that the finest frequency resolution was one of the following: ≤ ~ 4 Hz, ≤ ~ 2 Hz, ≤ ~ 1 Hz, ≤ ~ 0.5 Hz, ≤ ~ 0.25 Hz, or ≤ ~ 0.1 Hz. All statistical comparisons in subsequent sections were conducted as repeated-measures ANOVAs with post-hoc pairwise comparisons in SPSS software version 27.

Across adult and developmental datasets, the 4hz resolution level visibly shrunk the VEP waveform relative to the other parameters, suggesting worse fit for ERP data. In the 4-month data, the ≤ 2 Hz resolution level also resulted in altered VEP waveform morphology relative to the other wavelet levels and approaches (manual and automated segment rejection). The other resolution levels from ≤ 1 Hz to ≤ 0.1 Hz did not result in meaningful distortion of the ERP waveform relative to approaches without waveleting through initial visual inspection.

The effect of wavelet resolution level on trial retention rates was also considered across datasets. There were minimal differences between approaches for the adult VEP trial retention rates (F(7) = 2.18, p = 0.039, $ƞ_{p}^{2}$ = 0.086). No wavelet resolution level resulted in significantly different trial retention rates from the manually edited rate (all p > 0.05). Wavelet resolution levels from ≤ 1 Hz down to ≤ 0.1 Hz were not significantly different from each other in trial retention rates (all p > 0.05). This pattern of results suggests that waveleting approaches at most resolutions considered for optimization perform comparably each other and to the traditional manual editing approach for trial retention for low-artifact data. There were similarly minimal differences between resolution levels for both 4-month and 10-month developmental VEP trial retention rates (4-month: F(7) = 13.265, p = 2.5*10^-13^, $ƞ_{p}^{2}$ = 0.376; 10-month: F(7) = 5.736, p = 1.1*10^-5^, $ƞ_{p}^{2}$ = 0.112). As in the adult data, wavelet resolutions of ≤ 0.1 Hz, 0.25 Hz, and 0.5 Hz were not significantly different from each other in the number of retained trials in either developmental dataset (all p > 0.05). However, in both developmental datasets, manual editing resulted in significantly fewer retained trials compared to wavelet thresholded data at almost every resolution level (p < 0.05). This pattern of results suggests that waveleting across resolution levels does not impact trial retention meaningfully but outperforms the traditional manual editing approach for rates of trial retention in higher-artifact developmental data.

Finally, the degree of signal change during the wavelet-thresholding process was compared across wavelet resolution levels using the cross-correlation values for the EEG signal before and after wavelet-thresholding. There were striking and consistent effects of wavelet resolution level on the correlation of the EEG signal pre- and post-waveleting across adult and developmental datasets (adult: F(5) = 214.983, p = 3.98*10^-58^, $ƞ_{p}^{2}$ = 0.9; 4-month: F(5) = 822.939, p = 2.20*10^-85^, $ƞ_{p}^{2}$ = 0.743; 10-month: F(5) = 193.496, p = 3.69*10^-43^, $ƞ_{p}^{2}$ = 0.924). In each dataset, the more fine-grained the resolution, the less the EEG signal changed during wavelet-thresholding (significantly higher cross-correlation values were observed). Examining the cross-correlations at specific key frequencies in the signal confirmed that this effect was driven by change specifically in the low-frequencies as expected, since changing the wavelet resolution within the range we tested impacts treatment of the low frequency data. Notably, the finest resolution level of ≤ 0.1 Hz produced signal change the most comparable across frequencies, especially in the clean adult data with less low-frequency signal drift.

Given this pattern of results across waveform inspection, trial retention, and signal change during processing across datasets, the wavelet resolution level was set to ≤ 0.1 Hz to optimize wavelet-thresholding performance in ERP analyses.

**Table 3.** Average percent segments retained and correlation values pre- and post-waveleting in adult, 4-month, and 10-month data for wavelet level optimization.


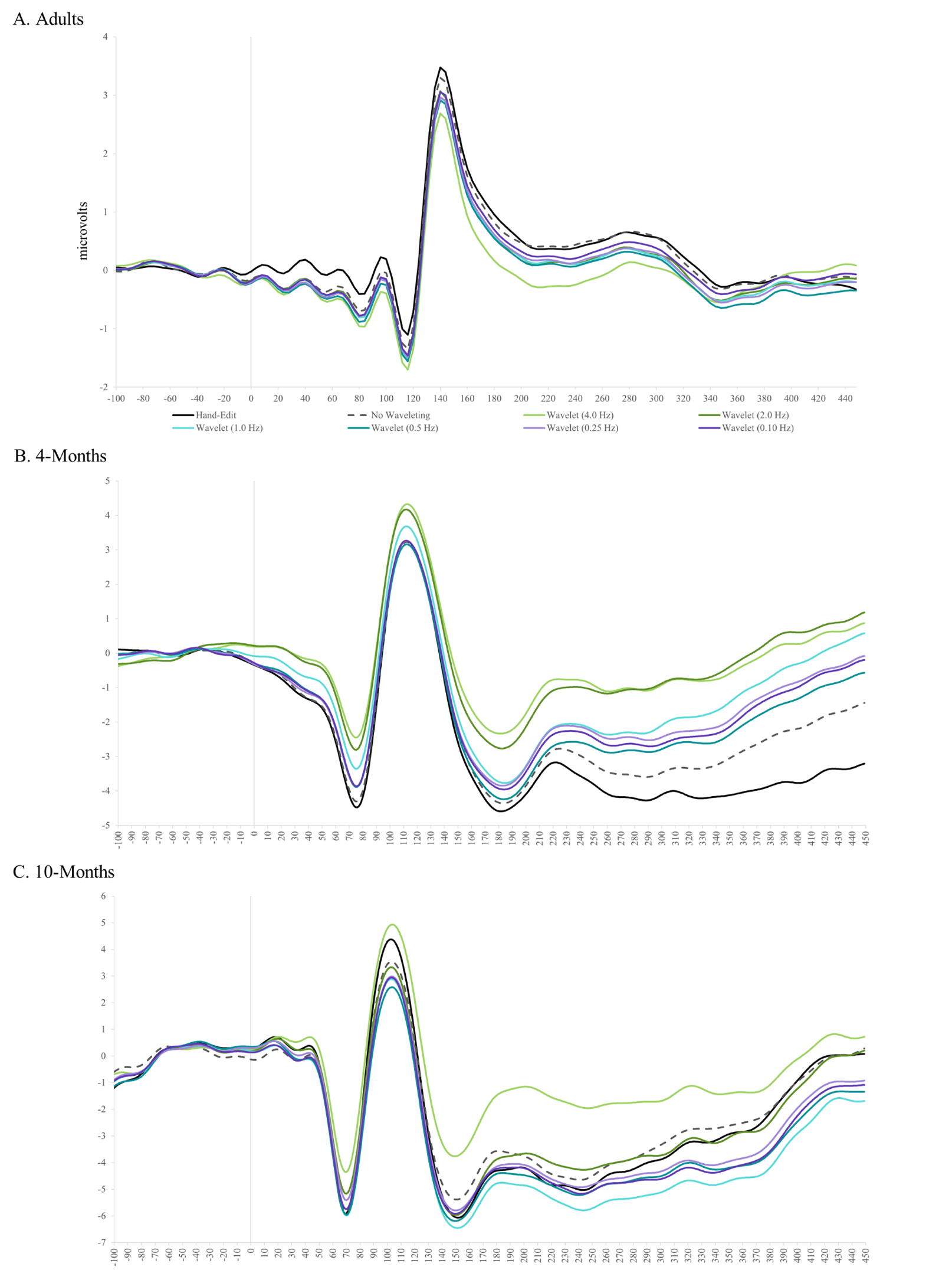


**Figure 2.** Three images illustrating the resultant VEP ERP waveform following processing using an array of wavelet levels, via hand-editing, and through the HAPPE+ER pipeline without artifact rejection steps, on adult (A), 4-month (B), and 10-month (C) data.
