# Supplementary material for: The HAPPE plus Event-Related (HAPPE+ER) software: A standardized preprocessing pipeline for event-related potential analyses
Source: Dev Cogn Neurosci. 2022 Jul 19;57:101140. doi: 10.1016/j.dcn.2022.101140 (PMC9356149; doi:10.1016/j.dcn.2022.101140)
Supplement: Supplementary material [file mmc2.docx]

### *Supplemental File 2*

### *Optimization of Independent Component Automated Rejection for Developmental ERP analyses*

For methods that use ICA, multiple algorithms exist that perform automated component rejection, but few have been tested or validated in developmental ERP data (though see iMARA (Haresign et al., 2021), Adjusted ADJUST (Leach et al., 2020), and ICLabel (Pion-Tonachini et al., 2017)). Therefore, we first compared across algorithms and rejection thresholds to optimize settings for ERP analyses in infant data using the 4-month VEP dataset. We selected the 4-month dataset for infant optimization instead of the 10-month dataset as the VEP is actively developing across all waveform components at 4 months of age and is more distinct from adult VEP morphology where prior validation exists. We compared the following automated rejection algorithms: Adjusted ADJUST, ICLabel, MARA, and iMARA. Adjusted ADJUST has previously been validated on infant data using an EGI high-density system as used for collecting the present datasets, so the default threshold settings from that validation were used here. For ICLabel, MARA, and iMARA algorithms without prior validation on the same system as this dataset, they were each evaluated at the standard rejection threshold of 0.5 or higher probability of being an artifact component and at a liberal threshold for data retention that only rejected components with 0.8 or higher probability of being artifact. Both the MARA (at both 0.5 and 0.8 thresholds) and the Adjusted ADJUST algorithms rejected multiple files (known to have usable VEP data as determined by manual inspection by an experienced researcher), so these algorithms were not considered further in primary analyses. Secondary analyses including Adjusted ADJUST on the subset of participants with retained data are referenced in brief and provided in the supplemental data tables. For primary analyses, the ICLabel 0.5, ICLabel 0.8, iMARA 0.5, and iMARA 0.8 algorithm-threshold combinations were compared statistically with manual editing and the segment rejection only approach in their effects on 1) participant and trial retention rates, 2) percent data retained post-processing, and 3) VEP morphology.

First, with respect to participant and trial retention, while all four algorithm-threshold combinations preserved 100% of the participants, there were significant differences in trial retention rates between these methods (F(5) = 13.491, p = 2.93*10^-10^, $ƞ_{p}^{2}$ = 0.380). All automated approaches retained more trials than the manual editing option (all p < 0.05), and ‘automated segment rejection only’ retained significantly fewer trials than iMARA 0.5, iMARA 0.2, and ICLabel 0.5 options (but was no different from ICLabel 0.8 in retention rates). The iMARA 0.5 and ICLabel 0.5 combinations retained significantly more trials than all other options (all p < 0.05) (but were not significantly different from each other, p > 0.05). Secondary analyses on the subset of participants retained by Adjusted ADJUST revealed that this algorithm retained significantly fewer trials than iMARA 0.2, iMARA 0.5, ICLabel 0.5, and ‘automated segment rejection only’ (all p < 0.05) and was not significantly different from trial retention rates of manual editing or ICLabel 0.8 (all p > 0.05). Thus, for trial retention, iMARA and ICLabel approaches both performed equally best with standard artifact thresholds (rather than liberal thresholds).

Second, the algorithm-threshold combinations were statistically compared on the percent data variance retained post-processing. This comparison did not include the manual editing or segment rejection only approaches since they do not remove variance from the continuous data prior to trial rejection. There was a statistically significant effect of algorithm-threshold combination on how much data was retained in the course of preprocessing (F(3) = 90.263, p = 2.588*10^-23^, $ƞ_{p}^{2}$ = 0.804). As expected, we found that both iMARA and ICLabel algorithms with liberal thresholds retained significantly more data variance post-processing than the algorithms with standard thresholds (all p < 0.05). Moreover, ICLabel 0.5 retained significantly more variance than the iMARA 0.5 option (p < 0.05). Thus, the liberal rejection thresholds did result in increased data retention during processing.

Finally, the effects of algorithm-threshold combinations were evaluated with respect to VEP peak amplitude morphology. Notably, the number of trials within an individual dataset used to generate the average VEP waveform affects the peak waveform amplitude (Luck, 2014), so for the following visualizations and statistical comparisons of VEP morphology, wherever automated methods had preserved greater numbers of trials relative to manual editing, a random subset of trials from each individual were selected to match the number of trials from manual editing for that same individual. There was a corresponding, consistent statistical pattern of algorithm-threshold effects on the VEP morphology across the N1, N1-P1 peak-to-peak, and P1-N2 peak-to-peak component amplitudes (N1: F(5) = 15.235, p = 2.33*10^-11^, $ƞ_{p}^{2}$ = 0.409; N1-P1: F(5) = 19.805, p = 4.94*10^-14^, $ƞ_{p}^{2}$ = 0.474; P1-N2: F(5) = 14.586, p = 5.9*10^-11^, $ƞ_{p}^{2}$ = 0.399). For each component amplitude, the manual rejection and automated segment rejection approaches were no different from each other but significantly larger than amplitudes from all MARA, iMARA, and ICLabel options (all p < 0.05). The iMARA and ICLabel approaches with the liberal 0.8 thresholds were no different from each other but were significantly larger than iMARA and ICLabel approaches with standard 0.5 thresholds (all p < 0.05). That is, while all component rejection approaches reduced ERP amplitudes relative to the traditional manual editing approach, the algorithms with liberal thresholds introduced less amplitude shrinkage (via retaining a greater percent of the data) than algorithms with standard thresholds for rejection.

This pattern of results suggests that iMARA and ICLabel achieve comparable performance across measures in developmental ERP analyses. However, automated component rejection performance, regardless of algorithm, depends highly on the rejection threshold selected. That is, there appears to be a tradeoff such that the standard rejection thresholds enable increased trial retention rates but remove a larger percent of the data and so result in more shrunken ERP amplitudes than the liberal rejection thresholds. Without a clear optimal algorithm-threshold option, we proceeded with the iMARA 0.2 combination for automated component rejection in the developmental datasets as this algorithm was one of the two better-performing options and was generated specifically for developmental ICA analyses.

**Table 4.** Statistics for the performance of various ICA rejection methods.


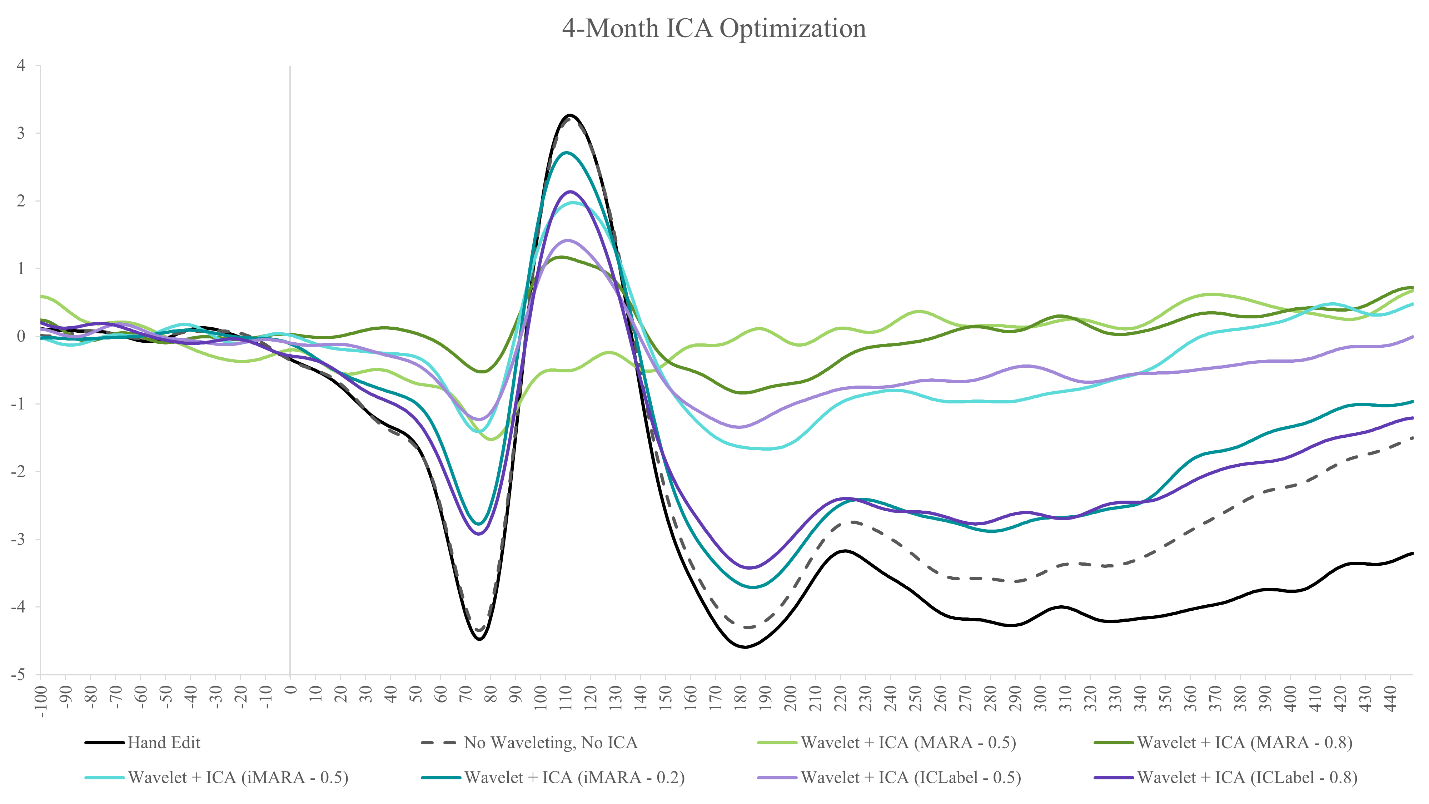


**Figure 3.** Figure illustrating the VEP ERP waveform generated after processing 4-month data using various ICA rejection methods, through hand-editing, and with segment rejection only. Greens indicate processing with MARA, blues with iMARA, and purples with ICLabel. Darker hues indicate a more liberal rejection threshold.
