# Supplementary material for: The HAPPE plus Event-Related (HAPPE+ER) software: A standardized preprocessing pipeline for event-related potential analyses
Source: Dev Cogn Neurosci. 2022 Jul 19;57:101140. doi: 10.1016/j.dcn.2022.101140 (PMC9356149; doi:10.1016/j.dcn.2022.101140)

Supplemental File 3

Electrode subset processed through HAPPE+ER (all colors), and the frontal ROI (blue), right temporal ROI (purple), and occipital ROI (darker green) used in various analyses.


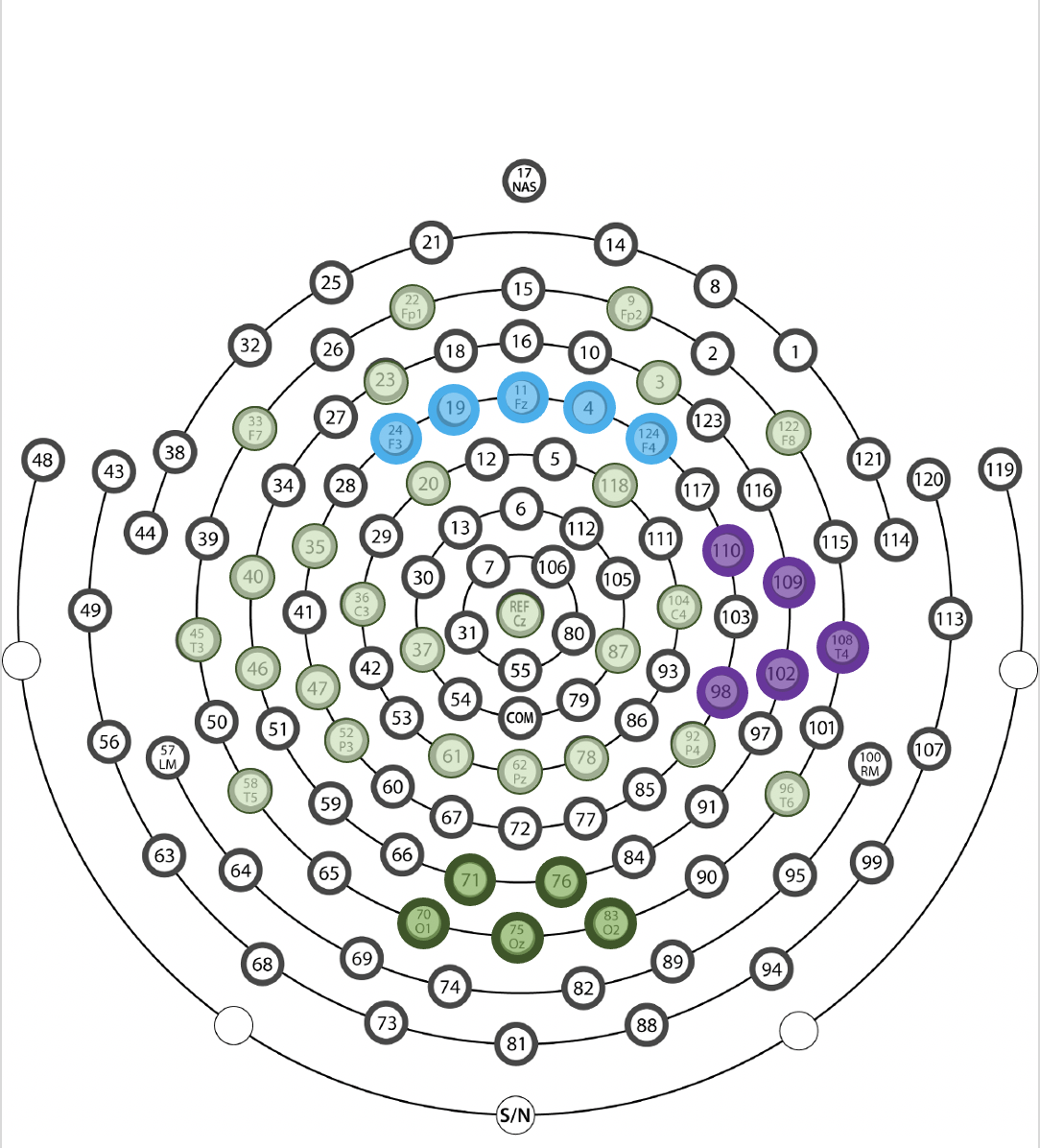

Supplement: Supplementary material [file mmc3.docx]
