# Supplementary material for: The HAPPE plus Event-Related (HAPPE+ER) software: A standardized preprocessing pipeline for event-related potential analyses
Source: Dev Cogn Neurosci. 2022 Jul 19;57:101140. doi: 10.1016/j.dcn.2022.101140 (PMC9356149; doi:10.1016/j.dcn.2022.101140)
Supplement: Supplementary material [file mmc4.docx]

Supplemental Analyses and Results with real developmental and adult EEG data

The artifact-rejection approaches were carried out as follows. W-ICA includes first performing an ICA decomposition of the EEG signal into components, after which all components' timeseries are subjected to wavelet transform and thresholded to remove artifact before all the components' cleaned timeseries are translated back to EEG channel data (Castellanos and Makarov, 2006). W-ICA followed by ICA includes a second ICA decomposition with automated component rejection (via MARA in adult data and iMARA in the developmental data). Wavelet-thresholding may also be employed independent of ICA, such that each channel’s timeseries undergoes thresholding for artifact instead of independent components from the ICA decomposition. Here we report wavelet-thresholding results with a soft threshold. Wavelet-thresholding with ICA includes initial wavelet thresholding followed by ICA with automated component rejection. ICA with automated component rejection may also be employed without waveleting. Finally, we examined removing all instances of wavelet-thresholding and ICA entirely (i.e., no artifact removal in continuous data) to match traditional preprocessing for ERP analyses. The data was subjected to either automated segment rejection (as for the prior approaches) or to manual segment rejection, carried out by an expert with over a decade of experience in manually editing EEG and ERP data. Single-expert rejection was used to align with typical (at least as reported) laboratory practices in data processing. The expert manually edited the data prior to the application of any automated approaches to avoid bias.

There are several important parameters that require further consideration when using ICA with ERP analyses. First, note that ICA typically requires a 1 Hz high-pass filter for adequate ICA performance (Winkler et al., 2015), which introduces a barrier to ERP analyses that include data below 1 Hz. To evaluate ICA within the context of ERP analyses, we took the following steps. First, each unfiltered file was copied, and the copy was subjected to a 1 Hz high-pass filter. Processing proceeded on the data copy for the approaches using W-ICA and/or ICA. As appropriate, the wavelet-thresholded artifact timeseries and/or the rejected artifact-laden independent component timeseries were then subtracted from the unfiltered file, effectively removing all waveleting- and/or ICA-identified artifacts from the data. The unfiltered file was then carried forward in analysis as the cleaned data eligible for further processing steps (e.g., ERP filtering, segmentation).

All statistical comparisons in subsequent sections were conducted as repeated-measures ANOVAs with post-hoc pairwise comparisons in SPSS software version 27.

### *Comparison of artifact correction approaches for ERP analyses: real ERP data*

We compared a wide array of approaches in real ERP datasets. We examined both adult ERP data (a dataset with very clean signals), as well as developmental ERP data at both 4- and 10-months of age (generally with higher levels of artifact contamination) from both healthy babies and those with complex medical histories including early general anesthesia experiences. Parental consent for infants and consent for adults was obtained in accordance with Boston Children’s Hospital’s IRB. All data were collected in a dimly-lit, sound-attenuated, electrically shielded room using 128-channel EGI Geodesic Sensor Nets with an online reference to Cz and NetAmps400 (MagStimEGI; Eugene, OR). Impedances were kept below 100 kilo-ohms in the infants and below 50 kilo-ohms in the adults in accordance with the impedance capabilities of the amplifier inside the shielded room.

Specifically, we evaluated a highly stereotyped and robust ERP across datasets, the visual evoked potential (Sokol, 1976; Varcin and Nelson, 2016). The adults (n = 24) underwent a grating-VEP paradigm with 126 trials successfully completed for each participant (mean file length: 379 seconds, standard deviation: 9 seconds, range: 366 – 403 seconds). The infants at both ages underwent a pattern-reversal VEP paradigm (n = 23 at 4-months, mean VEP trials completed: 103, standard deviation: 22, range: 73- 200 trials; n = 17 at 10-months, mean VEP trials completed: 108, standard deviation: 36, range: 64 – 232 trials). File lengths in the infant data were briefer and more variable than in the adults (4-months mean file length: 114 seconds, standard deviation: 49 seconds, range: 50 – 248 seconds; 10-months mean file length: 72 seconds, standard deviation 15 seconds, range: 53 – 112 seconds). Though the infant files are quite short in some cases, this is reflective of the kinds of data developmental studies routinely collect with young ages when paradigms must be brief given limited infant attention. To facilitate fair comparisons between wavelet-thresholding and ICA (whose performance in decomposing data into artifact and neural timeseries varies with file length and channel number, such that longer recordings enable better performance on large numbers of channels like the 128-channel system used here), we selected a spatially-distributed subset of 39 channels (See supplemental File 3) for analysis across all datasets, as we have previously noted adequate ICA decomposition with 39 channels (i.e. independent components) for files within this range of recording lengths (Gabard-Durnam et al., 2018). Each dataset was run through HAPPE+ER in batch with each of the continuous data rejection approaches above inserted into HAPPE+ER in place of wavelet-thresholding.

VEP components were then extracted as follows to compare the effects of different artifact correction approaches on their morphology. All files were segmented to include the first 450 milliseconds post-VEP stimulus onset and baseline corrected using a 100-millisecond baseline prior to the VEP stimulus within HAPPE+ER. To equate numbers of trials compared across approaches (as some approaches preserved more trials than others), a random subset of trials from each individual were selected to match the minimum number of trials preserved across approaches for that same individual. VEP component peak amplitudes were then calculated and extracted using HAPPE+ER’s generateERPs processing script across all datasets as follows. The mean VEP timecourse was calculated for each channel and averaged over a cluster of 5 occipital electrodes (O1, O2, E71, E75, E76) as the region of interest (ROI) to ensure a robust estimation of the VEP waveform across all ages. The peak amplitudes for the three VEP components, N1, P1, and N2 were then extracted from the occipital ROI using the following age-appropriate and paradigm-appropriate temporal windows in generateERPs. For infant datasets using a pattern-reversal VEP where there is high variability in the latencies of components, these windows were: N1 (30-120 msec), P1 (90 -160 msec), N2 (120-250 msec). For the adult dataset using a gradient VEP these windows were: N1 (80-148 msec), P1 (108-200 msec), N2 (160-300 msec). To correct for any differences in the N1 component’s peak amplitude that could drive subsequent differences in measuring the P1 and N2 peaks if absolute amplitudes were used, we then calculated peak-to-peak amplitude values for all components following the N1 by subtracting the component of interest from the prior component’s peak amplitude (i.e. P1 - N1 peak values, N2 - P1 peak values). These VEP morphology measures of interest are illustrated in Figure 3. These N1, N1-P1, and P1-N2 amplitude values were then subjected to statistical analyses across pre-processing approaches.


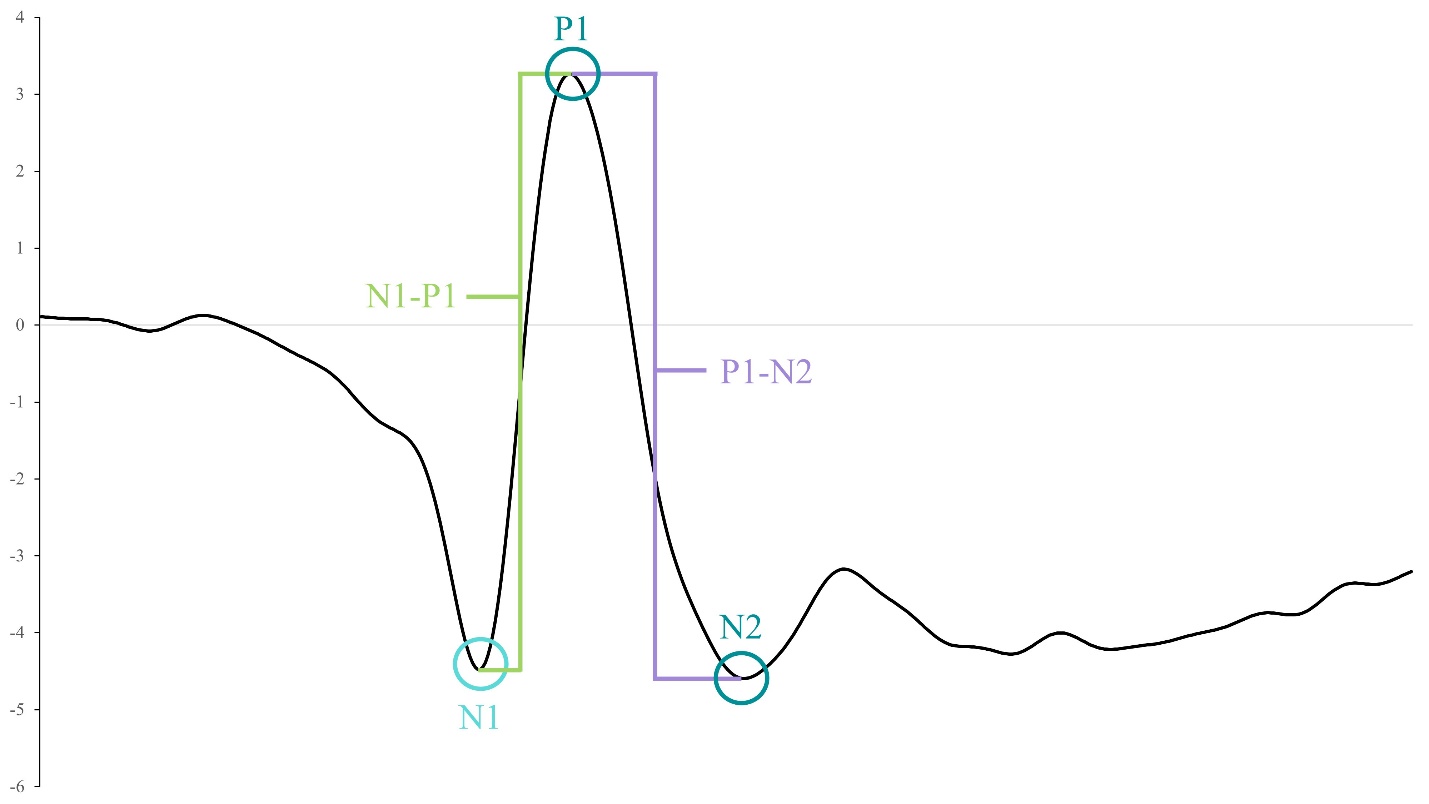


**Figure 3**. An image representing the different VEP components calculated for comparison of methods on ERP morphology. N1 is circled in light blue. N1-P1 is exemplified by the green lines indicating the difference between N1 and P1’s amplitudes. P1-N2 is exemplified by the purple lines indicating the difference between N1 and P1’s amplitudes.

Specifically, manual editing, automated trial rejection without prior artifact removal in the continuous signal, ICA, wavelet thresholding, wavelet thresholding with ICA, wICA, and wICA with ICA were compared using the following criteria to evaluate performance:

1. Rates of participant rejection. All files included in analyses were determined by an expert to have at least some sufficiently clean and usable trials, thus rates of rejecting entire files (i.e. all data or all trials removed) reflected un-necessary data attrition.
2. ERP morphology distortion. Specifically, using trial-matched ERPs generated across approaches, we evaluated whether the amplitude (peak and peak-to-peak amplitude magnitudes statistically compared) varied across methods for artifact correction relative to conditions without artifact correction, where no distortion would be expected (as no manipulations were performed on the data in the included trials) in the real data.
3. Degree of signal change pre- to post-correction and trial rejection (specificity/sensitivity of rejection).

In the absence of a ground truth signal, these measures together can indicate if an approach is removing neural with artifact signal (non-specific to artifact) or leaving artifact in the data (non-sensitive to artifact). For example, the adult dataset included in analyses contained largely clean data, so high degrees of signal change would indicate over-cleaning (non-specific signal removal). Relatively higher rates of trial rejection would indicate insufficient removal of the low-level of artifact in this dataset (leaving more artifact to be removed via segment rejection later). In the noisier developmental data, optimal performance should include lower rates of trial rejection indicating successful artifact removal (few bad trials left to reject) without over-cleaning the signal (which may be evident through significant amplitude shrinking in the ERP relative to other methods). High rates of trial rejection would indicate sub-optimal artifact removal (leaving high degrees of artifact during the artifact-correction step that must be removed during the later trial rejection step). High rates of signal change pre- to post-processing may indicate over-cleaning (non-specific signal removal). We note that signal removal can change signal amplitude (already observed for ICA), so to compare segment rejection across approaches without introducing a signal amplitude confound (e.g., approaches that shrink the amplitude will pass more trials through any given absolute voltage criteria for trial rejection), we used EEGLAB’s joint probability-based criteria for trial rejection (jointprob function, trials greater than 3 standard deviations different rejected as outliers).

Methods are compared below first for low-artifact adult VEP data and then in two higher-artifact developmental, clinical VEP datasets.

#### Adult ERP Comparisons

Participant rejection: The different artifact rejection methods were first compared across participant and trial retention rates in the adult VEP data. For all ICA-based approaches in the adult data, MARA with default settings was used for component rejection (components with artifact probability greater than 0.5 were removed). All methods retained 100% of the participants in the adult sample (data remained post-processing for all individuals). That is, there was no erroneous sample attrition regardless of processing strategy.

ERP Morphology distortion: Artifact rejection methods were evaluated on their impact on the adult VEP morphology using trial number matched datasets. In the context of this pristine dataset, all the automated artifact rejection approaches tested preserved gross morphology as determined by visual inspection (Figure 4, Supplemental File 4). However, repeated measures ANOVAs comparing the three VEP components’ peak values (N1, N1-P1 peak-to-peak, and P1-N2 peak-to-peak amplitudes) revealed a pattern of statistical differences between rejection methods (N1: F(6) = 8.338, p = 1.146*10^-7, $ƞ_{p}^{2}$ = 0.275; N1-P1: F(6) = 9.281, p = 1.754*10^-8^,$ƞ_{p}^{2}$ = 0.297; P1-N2: F(6) = 10.746, p = 1.037*10^-9^, $ƞ_{p}^{2}$ = 0.328). Specifically, the wavelet thresholding method and automated segment rejection approaches were not significantly different from each other (p > 0.05), but both generated a more negative N1 component amplitude than almost every other remaining method. Manual editing produced a significantly less negative N1 than wICA (in addition to waveleting and automated segment rejection), but was no different from ICA based approaches (p > 0.05). The ICA based approaches were no different from each other p > 0.05). Across the N1-P1 peak, automated segment rejection and wavelet thresholding were again no different from each other (p > 0.05). Automated segment rejection produced significantly larger N1-P1 amplitudes relative to all remaining approaches, while wavelet thresholding produced significantly larger amplitudes relative to all but manual editing (p > 0.05). Again, the ICA approaches were not significantly different from each other but significantly smaller than wavelet thresholding, automated segment rejection, and manual editing. For the P1-N2 component, wavelet thresholding, automated segment rejection, manual editing, and wICA were not significantly different from each other but all significantly larger in amplitude than the ICA options. This pattern of results in the clean adult data suggests that the ICA-based approaches employed here reduced ERP amplitudes, while the wavelet-thresholding and automated segment rejection approaches produced the largest VEP amplitudes comparable to the manual editing condition.

Signal change and trial retention: Finally, while there was also a significant omnibus test for trial retention across approaches in this very clean dataset (F(6) = 23.99, p = 2.59*10^-19^, $ƞ_{p}^{2}$ = 0.511), this was driven by the manual editing and automated segment rejection approaches retaining significantly fewer trials than all of the wavelet or ICA based approaches (which were no different from each other statistically). This pattern suggests that a) artifact correction approaches can improve trial retention even in clean, adult samples, and b) no artifact correction approach was grossly insensitive to the low level of artifact present in the data (which would be indicated by significantly higher rates of trial rejection to remove remaining artifact after the artifact correction step). However, the ICA-based methods all retained less than 50% of the data post-processing (Table 5). Correspondingly, the ICA-based methods demonstrated significantly lower correlations between the pre- and post-processed data compared to waveleting-based approaches across frequencies (F(4) = 66.625, p = 2.37*10^-26^, $ƞ_{p}^{2}$ = 0.743). There were no significant differences within the ICA-based methods (ICA, waveleting with ICA, wICA with ICA) in the correlations pre- and post-processing (all p > 0.05). There were no significant differences within the waveleting approaches (wavelet thresholding, wICA) in the correlations pre- and post-processing either (p > 0.05). Taken together, these metrics in the clean adult data suggest the wavelet-based methods preserved substantially more of the underlying neural signal during pre-processing compared with ICA-based approaches, which may over-reject neural data with the artifact signal in this fairly clean data context (i.e. non-specific signal rejection).

Together this pattern of results in the low-artifact adult VEP data demonstrates that of the automated artifact correction/rejection approaches, the wavelet thresholding method and automated segment rejection best-preserved VEP morphology (larger component amplitudes) and the EEG signal during pre-processing (higher correlations pre- to post-processing relative to ICA approaches, ICA retaining less than 50% signal), without retaining excess artifact (no significant differences in trial rejection rates). Notably, this adult dataset was intentionally particularly pristine (experienced EEG researchers were included in the sample and the paradigm was brief). Still, there was an observed benefit here to wavelet-thresholding relative to the ‘segment rejection only’ approach (no artifact correction in continuous data prior to trial rejection), indicating that artifact correction in continuous data should provide benefits in adult ERP data more broadly. These results across evaluation metrics also suggest wavelet-thresholding provides the best automated artifact correction approach of those tested here for the adult VEP data.


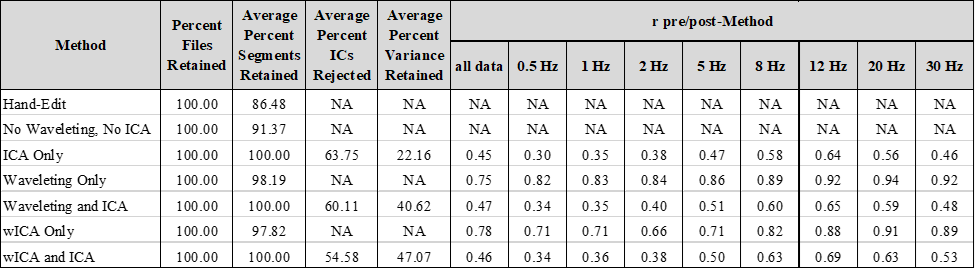


**Table 3.** Statistics for the performance of various artifact rejection methods on adult data.

Developmental ERP Comparisons

The different artifact rejection methods were also evaluated on their impact on two developmental VEP datasets from 4- and 10-month-olds that included more artifacts during recording than the healthy adult dataset. For all ICA-based approaches in the developmental data, the infant-trained iMARA algorithm with the liberal data-retention setting was used for component rejection (components with artifact probability greater than 0.8 were removed). As before, methods were evaluated on their effects on the three defined performance criteria, elaborated below.

*Participant rejection*: All methods tested on both 4- and 10-month datasets retained 100 percent of the participants. That is, there was no erroneous sample attrition regardless of processing strategy.

*ERP Morphology distortion*: Next, methods were compared with respect to effects on VEP morphology across 4- and 10-month datasets using trial number matched datasets (matched to manual editing trial retention values) (Figure 4). In the 4-month dataset there was a consistent pattern of effects across VEP components (N1: F(6) = 5.253, p = 7*10^-5^, $ƞ_{p}^{2}$ = 0.193; N1-P1: F(6) = 12.933, p = 1.83*10^-11^, $ƞ_{p}^{2}$ = 0.370; P1-N2: F(6) = 9.191, p = 2.09*10^-8^, $ƞ_{p}^{2}$ = 0.295). Specifically, waveleting methods (wavelet thresholding and wICA) resulted in amplitudes that were not significantly different from those produced by either manual editing or automated segment rejection without prior artifact removal (p > 0.05; except for P1-N2 amplitudes after waveleting approaches were significantly smaller than manual editing amplitudes but not significantly different from those achieved by automated segment rejection only). In contrast, ICA-based approaches almost always resulted in smaller amplitudes compared to manual editing and automated segment rejection (except wICA with ICA N1 component was not significantly different from manual editing, p = 0.07). There was less differentiation between methods in the 10-month data sample. The omnibus F test was not significant for the test of differences in the N1 peak amplitude across methods (F(6) = 1.459, p = 0.2, $ƞ_{p}^{2}$ = 0.084) or for the N1-P1 peak-to-peak amplitudes (F(6) = 1.733, p = 0.121, $ƞ_{p}^{2}$ = 0.0.098). P1-N2 peak-to-peak amplitudes were significantly different across methods (F(6) = 3.009, p = 0.010, $ƞ_{p}^{2}$ = 0.158). Namely, manual editing produced significantly larger peak-to-peak amplitudes than all other approaches except wICA and wICA with ICA (p > 0.05). Automated approaches were not significantly different from each other.

*Signal change and segment retention*: A statistically significant effect of artifact rejection method on trial retention rate was observed in both the 4- and 10-month datasets (4-month: F(6) = 29.275, p = 4.23*10^-22^, $ƞ_{p}^{2}$ = 0.571). Specifically, in the 4-month data, automated segment rejection retained fewer trials than all other approaches (all p < 0.05), and manual editing retained significantly fewer trials than all other methods except wICA (no different) and automated segment rejection (manual editing retained significantly more trials p = 0.007). Wavelet thresholding preserved significantly more trials than manual editing (p = 0.026), automated segment rejection (p = 6.45 *10^-8^), and wICA (p = 0.036), was no different than wICA with ICA (p = 0.08), and kept significantly fewer trials than ICA (p = 0.015) and waveleting with ICA (p = 0.001). That is, in the 4-month data, there was a clear trial retention benefit to using automated correction approaches compared to manual rejection or automated segment rejection without artifact correction. However, the interpretation of the segment retention results amongst the automated artifact correction strategies is not straightforward as the trial rejection strategy was based on signal amplitude thresholds, but we have already noted differences in signal amplitude in the 4-month data for ICA compared to wavelet approaches (see VEP morphology results above). Therefore, it is difficult to say if ICA approaches retained more segments in this dataset because artifact was more appropriately removed or because the entire signal’s amplitude was reduced significantly and thus more likely to pass the amplitude threshold for inclusion. Fortunately, the 10-month results offer additional information as we did not observe differences in signal amplitude as a function of artifact correction approach for this dataset.

There was also a statistically significant effect of artifact rejection method on trial retention rate was observed in the 10-month datasets (F(6) = 15.023, p = 4.72*10^-12^; $ƞ_{p}^{2}$ = 0.484). In the 10-month data, wavelet thresholding preserved significantly more trials than all other methods (all p < 0.05). Manual editing and automated segment rejection preserved significantly fewer trials than all artifact correction methods (p < 0.05, not significantly different from each other p = 0.673). ICA approaches were not significantly different from each other in terms of trial retention rates (but retained significantly fewer trials than wavelet-thresholding). As in the 4-month data, a trial retention benefit to using automated artifact correction strategies was observed (instead of manual editing or automated segment rejection only). In the 10-month data where VEP amplitudes were largely equivalent between artifact correction strategies, wavelet-thresholding outperformed every other method in retaining more trials.

Across developmental and adult datasets, these results suggest several trends, though further testing is required to draw conclusions across a range of ERPs. First, across datasets, wavelet-thresholding generally better preserved VEP amplitudes relative to the other automated artifact correction approaches, resulting in amplitudes consistent with the current field-standard from manual-editing (whereas ICA approaches reduced VEP amplitudes in two of the three datasets). Second, these results also illustrate the difficulty of testing methods and interpreting results using only real ERP data without known ground-truth signal. Although the 4-month sample results were somewhat confounded across approaches in terms of morphology changes and trial retention results, in the 10-month dataset wavelet-thresholding returned equivalent VEP morphology but retained significantly more trials than all other approaches including ICA. In the adult data, wavelet-thresholding returned equivalent trials relative to the artifact correction strategies (with all strategies returning almost all trials in this clean dataset), and preserved VEP amplitudes better than ICA-based artifact correction strategies when compared to manual editing and automated trial rejection without prior artifact correction. Though this overall pattern of results suggests wavelet thresholding is a promising approach for ERP preprocessing, we advise the reader to primarily consider the simulated ERP results within the main manuscript.


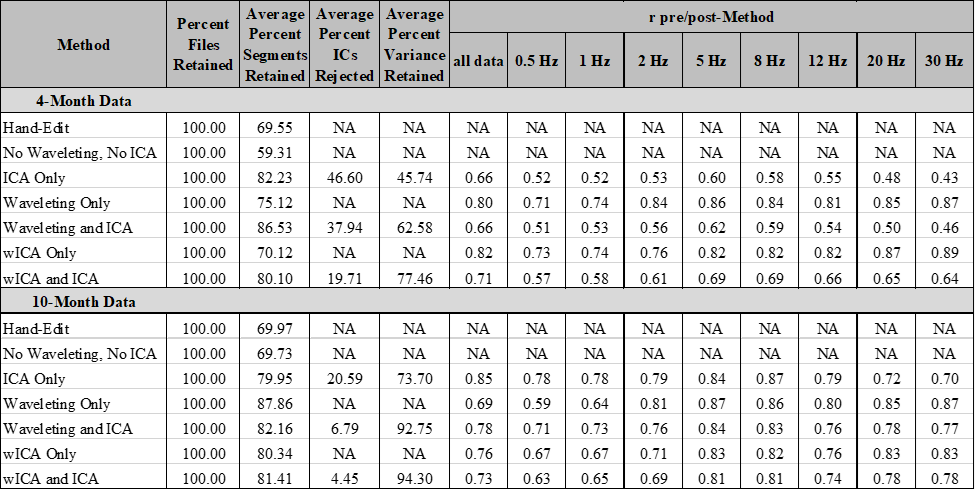


**Table 4.** Statistics for the performance of various artifact rejection methods on 4-month and 10-month data.


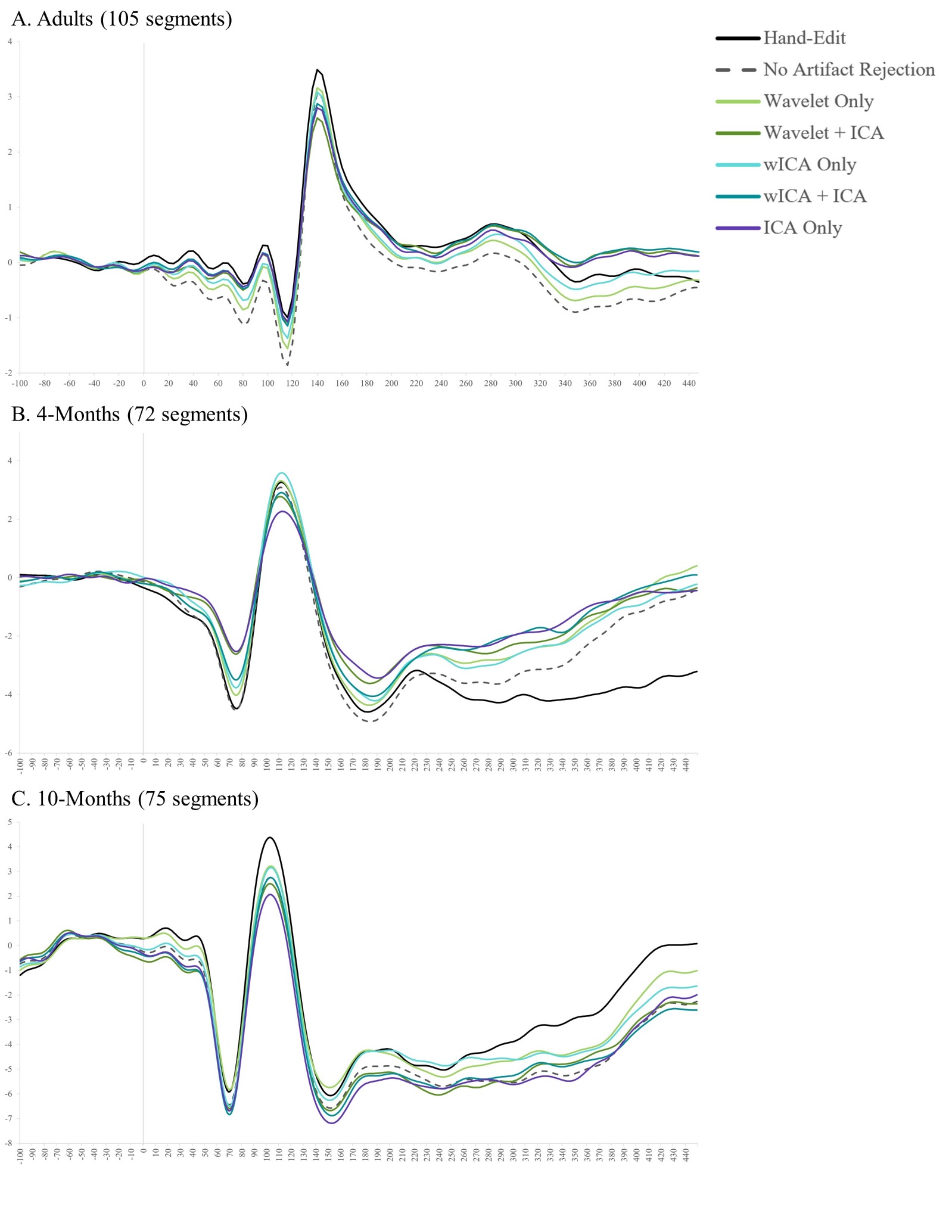


**Figure 4.** Three images illustrating the resultant VEP ERP waveform following processing using an array of artifact rejection methods and segment rejection only, on adult (A), 4-month (B), and 10-month (C) data.
